# Enhancing Stability and Performance in Perovskite Solar Cells through Rationally Designed Phenanthro[9,10‑*d*]imidazole Derivatives for Tailored Interfacial Engineering

**DOI:** 10.1021/acsami.5c22843

**Published:** 2026-01-20

**Authors:** Rajarathinam Ramanujam, Zhong-En Shi, Chien-Yu Lung, Gebremariam Zebene Wubie, Sie-Rong Li, Chih-Ping Chen, Shih-Sheng Sun

**Affiliations:** † 38017 Institute of Chemistry, Academia Sinica, Nankang, Taipei 11529, Taiwan, Republic of China; ‡ Taiwan International Graduate Program, Sustainable Chemical Science and Technology, Academia Sinica, Nankang, Taipei 11529, Taiwan, Republic of China; § Department of Applied Chemistry, National Yang Ming Chiao Tung University, Hsinchu 30050, Taiwan, Republic of China; ∥ Department of Materials Engineering and Organic Electronics Research Center, 56082 Ming Chi University of Technology, 84 Gunjuan Road, Taishan, New Taipei City 24301, Taiwan, Republic of China; ⊥ College of Engineering and Center for Sustainability and Energy Technologies, Chang Gung University, Taoyuan City 33302, Taiwan, Republic of China

**Keywords:** perovskite solar cell, phenanthro[9,10-*d*]imidazole, interfacial layer material, nickel oxide, passivation, dopant-free, inverted device, thermal stability

## Abstract

Interfacial engineering plays a significant role in advancing the performance and stability of perovskite solar cells (PSCs). In inverted PSCs, nickel oxide (NiO_*x*_) is widely used as a hole transport material (HTM). However, poor interactions at the NiO_*x*_/CH_3_NH_3_PbI_3_ (MAPbI_3_) interface often lead to reduced device stability and power conversion efficiency (PCE). To address this issue, dopant-free ultrathin interfacial layers (IFLs) have been introduced between NiO_*x*_ and the perovskite layer to enhance the interfacial interactions and optimize the device performance. In this work, phenanthro­[9,10-*d*]­imidazole derivatives, SR-1 and SR-2, were rationally designed and synthesized as IFL materials in p–i–n devices with a device configuration of indium tin oxide (ITO)/NiO_*x*_/IFL/MAPbI_3_/PCBM/BCP/Ag. These IFLs not only modify the energy levels of NiO_*x*_ but also improve the surface morphology and crystallinity of MAPbI_3_, effectively passivate interfacial defects, facilitate charge extraction, and reduce trap density at the NiO_*x*_/MAPbI_3_ interface. As a result, the PCEs of both devices with SR-1 and SR-2 IFLs outperformed those of the pristine device. The best performance of 20.3% efficiency with nearly negligible hysteresis was achieved from the device with SR-1. Furthermore, the devices with SR molecules achieved remarkable thermal stability under continuous heating at 60 °C and 50–60% relative humidity, highlighting the potential of SR-based IFLs for stable and efficient PSCs.

## Graphical abstract

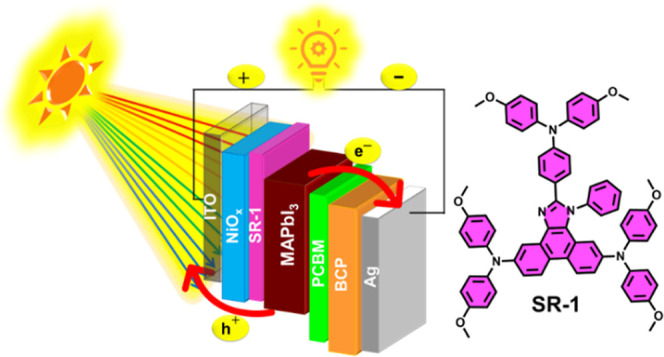

## 1 Introduction

Organometallic halide perovskites have emerged as promising materials for next-generation photovoltaic technology.^1,2^ Their cost-effectiveness and ease of fabrication process position perovskite solar cells (PSCs) as potential rivals to currently available silicon-based solar cells.^3^ Key advantages of perovskites, including a long charge carrier diffusion length, high charge carrier mobility, and strong light absorption ability, render them highly efficient light-harvesting materials in PSCs.^4,5^ PSC power conversion efficiency (PCE) has continuously grown from 3.8% in 2009 to 26.95% and may eventually surpass the 31% theoretical maximum predicted by the Shockley–Read–Hall theory.^6–12^ Among PSC architectures, the inverted planar device configuration (p–i–n) has garnered significant attention due to its simplicity, long-time stability, low hysteresis, cost-effective fabrication process, and compatibility with tandem solar cells.^13^ The hole transport material (HTM) is deposited onto the indium tin oxide (ITO) substrate by using a low-temperature solution processing protocol for making p–i–n PSCs. These HTMs play the role of charge extraction and hole transport upon light absorption and serve as a protective layer for the perovskite from moisture, oxygen, and other factors leading to device degradation.^14^

Two common organic polymers, poly­(bis­(4-phenyl)-(2,4-dimethyl phenyl)­amine (PTAA) and poly­(3,4-ethylenedioxythiophene):poly­(styrenesulfonate) (PEDOT:PSS), are widely used as HTMs for p–i–n PSCs. High-performance PSCs have been achieved through modification of PTAA and PEDOT:PSS.^15^ In particular, introducing ultrathin PTAA layers effectively achieved defect passivation and led to efficient and stable PSCs with high fill factor (FF) and eliminated hysteresis.^16^ Nevertheless, developing new low-cost HTMs to mitigate nonradiative recombination losses,^17^ replace expensive PTAA, and overcome perovskite film degradation caused by the intrinsic hygroscopic nature of PEDOT:PSS is still an ongoing research challenge in p–i–n PSCs.^18^ On the other hand, inverted PSCs utilizing inorganic materials, including NiO_*x*_, CuSCN, and VO_*x*_, as HTMs have achieved decent performance.^19^ In particular, when compared to other inorganic and polymer-based HTMs, NiO_*x*_ offers notable semiconductor features, including a wide bandgap, suitable energy level, and outstanding thermal and chemical stability.^20,21^ Moreover, the fabrication of NiO_*x*_-based p–i–n PSCs can be achieved at low temperatures through solution processing methods, significantly reducing the energy cost for production. However, many defects and trap sites exist in NiO_*x*_ prepared from the low-temperature solution process that greatly hamper the perovskite nucleation deposited on top of the NiO_*x*_ layer. The low crystallinity and grain size of the perovskite layer result in poor hole extraction and unfavorable carrier recombination.^22,23^ Thus, effective interfacial engineering to improve the interactions between NiO_*x*_ and perovskite layers is highly desired to achieve high-performance PSCs.

The Ni^3+^ cation sites within the NiO_*x*_ film can behave as either Lewis electron acceptors or Bronsted proton acceptors. Excess Ni^3+^ cations within the NiO_*x*_ film, commonly known as defect sites, may introduce unfavorable redox reactions within the perovskite film and degradation of the MAPbI_3_ to uncoordinated Pb^2+^ and I^–^ ions, which leads to significant carrier recombination, high charge extraction barriers, and small grain size at the NiO_*x*_/perovskite interface.^24,25^ However, the low intrinsic p-type electrical conductivity of NiO_*x*_ can lead to increased series resistance in devices.^37^ Thus, it is necessary to maintain an optimal ratio of Ni^3+^/Ni^2+^ to ensure the p-type characteristics of NiO_*x*_ and avoid unfavorable redox reactions.^26,27^

Recently, numerous studies have been reported focusing on interfacial engineering of defect passivation and their associated challenges at the NiO_*x*_/perovskite interfaces.^28^ These studies play crucial roles in understanding PCE enhancement and the long-term stability of PSCs in ambient environments. Notable examples in interfacial engineering of defect passivation include in situ surface modification of NiO_*x*_ by using potassium borohydride,^29^ urea,^30^ nickel nitrate,^31^ copper metal doping on NiO_*x*_,^32^ and alkali halides (NaCl or KCl).^33^ Molecules containing heteroatoms (N, P, O, S) with Lewis base properties have been incorporated as interfacial layer (IFL) materials between the NiO_*x*_/MAPbI_3_ interface.^34^ Regarding the IFL materials, organic small molecules (OSMs),^35^ organic self-assembled monolayers,^36^ and organic polymers^37^ have been explored in improving the properties of the NiO_*x*_/MAPbI_3_ interface in NiO_*x*_-based p–i–n PSCs.

Among these materials, OSMs hold diverse advantages for device fabrications. The simple and low-cost synthetic procedures, facile structure modification, and thus tunability of the energy levels and property compatibility with NiO_*x*_ and perovskite are superior characteristics for OSMs as IFL materials. Nevertheless, OSMs have been explored relatively less compared to other materials, such as IFLs in inverted PSCs. Examples of OSMs used as IFLs in inverted NiO_*x*_/MAPbI_3_-based PSCs, prepared from various organic core moieties, include triphenylamine-imidazole,^38^ dibenzofulvene,^39^ carbazoles,^40^ phenothiazine,^41^ helicene,^42^ tetraphenylethylene,^43^ and truxene,^44^ By effectively passivating the intrinsic defects at the NiO_*x*_/MAPbI_3_ interface via the formation of Lewis acid/base adducts. These OSMs can tune the energy levels of NiO_*x*_ and its p-type conductivity to enhance the hole mobility.^45^

Phenanthro­[9,10-*d*]­imidazole (PIM) derivatives are widely utilized as semiconductors in optoelectronic applications.^46^ The core of a PIM consists of a phenanthrene fused with an imidazole ring, resulting in a rigid and planar structure. The energy levels can be tuned by incorporating peripheral amines at the PIM terminal positions, which is suitable for fabricating solution-processable semiconducting layers for optoelectronic applications.^47^ In addition, the imidazole ring contains pyridine- and pyrrole-like amphoteric nitrogen atoms. The strong Lewis basicity of the pyridine-like nitrogen atoms produces strong interactions with the uncoordinated Lewis acidic Pb^2+^ ions, forming Lewis adducts. This interaction is highly effective in passivating intrinsic defects, minimizing carrier recombination, and promoting a smooth morphology at the HTM/MAPbI_3_ interface.^48^ It is worth noting that imidazole-based derivatives are rarely employed as HTMs in PSCs. Decent device performance has been reported for imidazole-based derivatives used in both conventional n–i–p and inverted p–i–n PSCs.^49,50^ A study on functionalizing the C6/C9 positions of PIM-based OSMs as HTMs in PSCs indicated that the PCE of the n–i–p device reached up to 21.65%, while the PCE of the inverted p–i–n device was slightly lower at 19.11%.^51^

Herein, two T-shaped SR molecules, SR-1 and SR-2, were designed and synthesized via conventional C–N and C–C coupling reactions. The central core of PIM was integrated with either *para*-methoxydiphenylamine (*p*-OMeDPA) or *para*-methoxytriphenylamine (*p*-OMeTPA) at the various positions of C2, C5, and C10. SR molecules deposited as an ultrathin layer on NiO_*x*_ HTM not only tuned the valence band energy of NiO_*x*_ but also slightly enhanced the ratio of Ni^3+^ vacant sites within the electronic state of the nickel element. More importantly, SR molecules can passivate the perovskite layer’s defect sites (uncoordinated Pb^2+^), leading to enhanced hole mobility and lower defect state density. The PSCs with a p–i–n device configuration of ITO/NiO_*x*_/w/or w/o SRs/MAPbI_3_/PCBM/BCP/Ag were fabricated. The SR molecules worked as charge-transporting IFLs at the NiO_*x*_ and perovskite interface. As a result, enhanced charge extraction, efficient defect passivation, and reduced recombination were achieved at the NiO_*x*_/perovskite interface. SR-1-derived PSC achieved an efficiency of 20.3%, which is much improved compared to the control device’s efficiency (17.4%). The PCEs of the current work are the highest among the reported works based on the imidazole HTMs for the inverted type of PSCs (Table S1). In addition, SR-passivated devices achieved remarkable thermal stability under unencapsulated conditions with continuous heating at 60 °C and 50–60% relative humidity. For clearer benchmarking, Table S2 compares our results with recent NiOx-based PSCs incorporating small-molecule, SAM-based, and polymeric interfacial layers. Although some advanced systems report efficiencies above 22–26%, these typically rely on complex anchoring motifs or multicomponent passivation strategies.

## 2 Results and Discussion

The molecular structures of SR molecules are shown in Figure 1a, and their synthetic pathways are presented in Scheme 1. The starting material of 5,10-dibromo-2-(4-bromophenyl)-1-phenyl-1*H*-phenanthro­[9,10-*d*]­imidazole was synthesized through a one-step multicomponent cyclization.^46^ Subsequently, SR-1 and SR-2 molecules were synthesized through conventional Buchwald–Hartwig C–N coupling reactions and Suzuki–Miyaura C–C coupling reactions by compound 2 and *p*-OMeDPA or *p*-OMeTPA, respectively. The purity and identity of all newly synthesized molecules were confirmed by high-resolution mass spectrometry and ^1^H NMR and ^13^C NMR spectroscopy (see Figures S1–S8 in Supporting Information).

**Figure 1:**
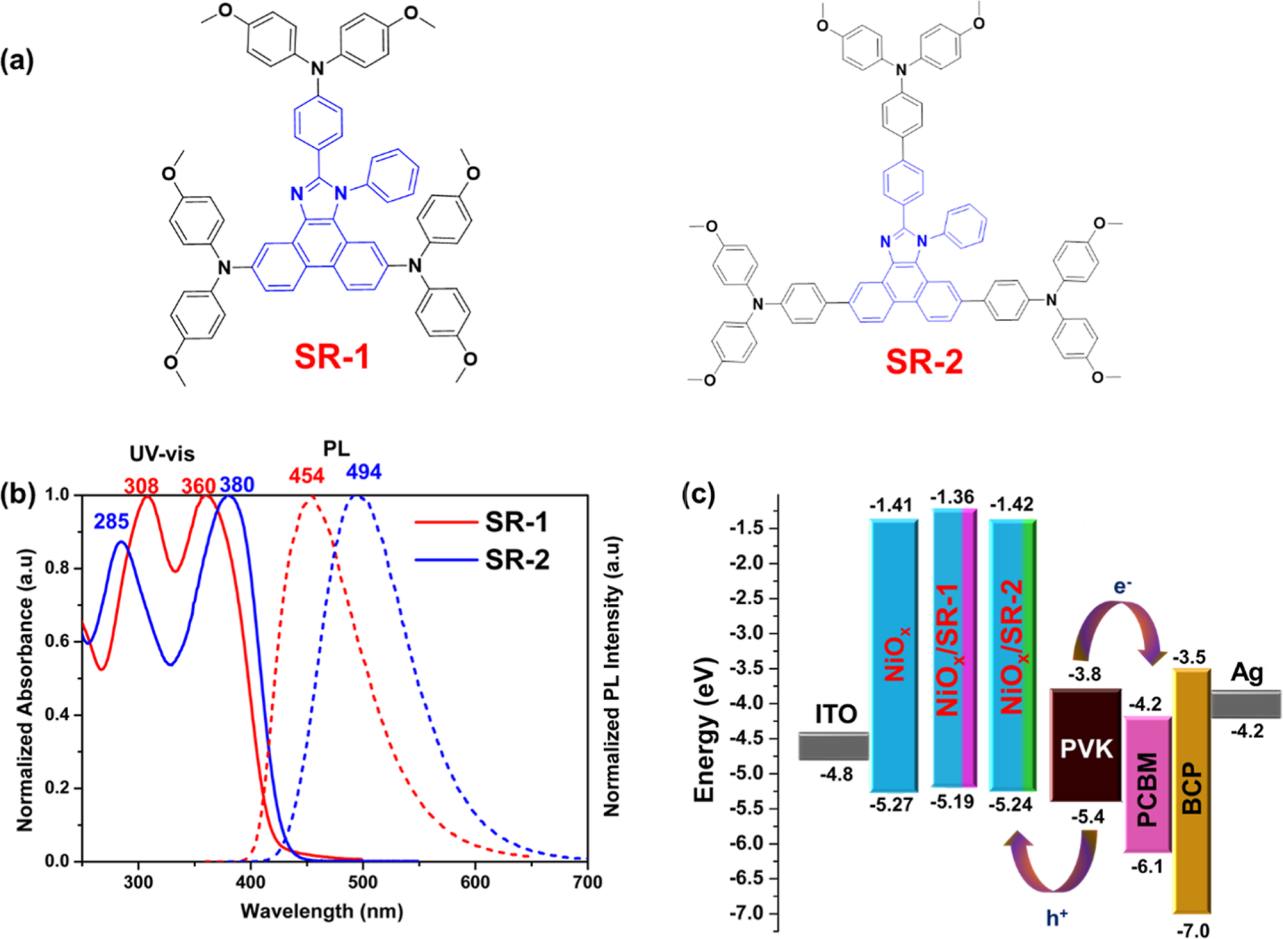
(a) Chemical structures of SR molecules; (b) normalized UV–vis absorption and fluorescence spectra of SR molecules; (c) the energy levels of SR molecules embedded between NiO_*x*_ and perovskite in the p–i–n device architecture.

**Scheme 1:**
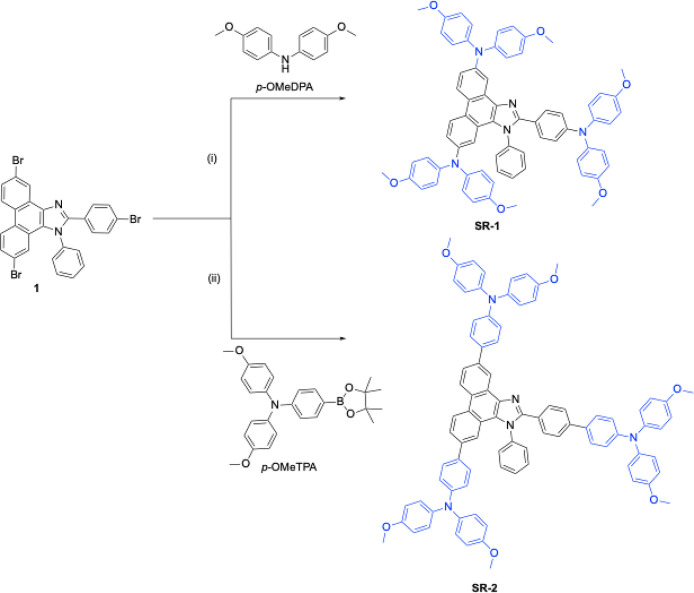
Synthetic Routes of SR-1 and SR-2. (i) Pd_2_(dba)_3_, [(^*t*^Bu)_3_PH]­BF_4_, ^*t*^BuONa, Toluene, 110 °C, 66%. (ii) Pd­(PPh_3_)_4_, K_2_CO_3_, Toluene, 110 °C, 55%

To better understand the optical properties, the UV–vis absorption and fluorescence spectra of SR molecules were measured in dichloromethane (DCM) solutions, as shown in Figure 1b. The corresponding spectral data are listed in Table 1. SR-1 and SR-2 show similar absorption spectral profiles. The extended π-conjugation linkage in the SR-2 molecule leads to a red shift in the low-energy absorption maxima compared to SR-1. A similar bathochromic shift in fluorescence of SR-2 was also observed compared to that of SR-1.

**Table 1:** Optical Properties and Energy Level Analysis of SR Molecules

OSM’s	λ_abs_ (nm)^a^	λ_f_ (nm)^a^	*E*_VB_ (eV)^b^	*E*_g_ (eV)^c^	*E*_CB_ (eV)^d^
SR-1	308, 360	454	–5.19	–3.83	–1.36
SR-2	285, 380	494	–5.24	–3.82	–1.42

a The measurements were conducted in DCM solutions at a concentration of 1 × 10^–5^ M. λ_abs_ is the UV–vis absorption maximum, and λ_f_ is the fluorescence maximum wavelength.

b ^,^ The valence band levels (*E*_VB_) and band gaps (*E*_g_) were calculated after depositing SR molecules on top of the ITO/NiO_*x*_ substrate.

c The conduction band levels (*E*_CB_) were calculated using the equation *E*_CB_ = *E*_VB_ + *E*_g_.

The electrochemical properties of SR-1 and SR-2 were studied by using cyclic voltammetry (CV) and differential pulse voltammetry (DPV) in DCM solutions (Figure S9). Both compounds exhibit multiple quasi-reversible oxidation waves, attributed to redox processes at the peripheral amino groups. The first oxidation potentials obtained from DPV were used to estimate the HOMO levels, yielding −4.8 eV for SR-1 and −5.0 eV for SR-2. Combined with optical bandgaps derived from the intersections of their absorption and emission spectra, the corresponding LUMO levels were calculated to be −1.8 eV and −2.1 eV for SR-1 and SR-2, respectively.

The thermal properties of SR-1 and SR-2 were evaluated by thermogravimetric analysis (TGA) and differential scanning calorimetry (DSC). Figure S10­(a) shows the TGA curves with decomposition temperatures (*T*_d_) at 433 and 442 °C for SR-1 and SR-2, respectively. Figure S10­(b) shows the DSC curves with the glass transition temperature (*T*_g_) observed at 164 and 170 °C for SR-1 and SR-2, respectively. The high *T*_g_ values of both SR molecules are beneficial for fabricating pinhole-free perovskite films and ensuring thermal stability under PSC operating conditions.^52^

Ultraviolet photoelectron spectroscopy (UPS) measurements were conducted to evaluate the electronic energy band alignment of NiO_*x*_ in the presence of the SR molecules (Figure S11). The binding energy (BE) values found from cutoff and onset regions are used to calculate the *E*_VB_ values according to the equation *E*_VB_ = 21.22 – (*E*_cutoff_ – *E*_onset_).^15^ Figure 1c shows the device configuration and the energy levels (see Tables 1 and S3). After the SR molecules are introduced, the VB levels are slightly upshifted, with the VB from −5.27 eV for ITO/NiO_*x*_ to −5.19 and −5.24 eV for ITO/NiO_*x*_/SR-1 and ITO/NiO_*x*_/SR-2, respectively. The SR-1- and SR-2-modified substrates exhibit 80 and 30 meV upshifting of the VB level, respectively, compared to the pristine NiO_*x*._ The additional driving force is expected to further enhance the charge extraction at the interface between the NiO_*x*_/SR and the perovskite layer. The observed alterations in *E*_VB_ suggest that the SR molecules modified the electronic state of the NiO_*x*_ surface. This phenomenon has been substantiated through X-ray photoelectron spectroscopy (XPS) analysis (vide infra). Meanwhile, the energy level of NiO_*x*_ films has been determined both with and without the layer of SR molecules, utilizing the conventional Tauc plot method derived from the absorption spectra, as depicted in Figure S12, and the data are collected in Table 1. The *E*_g_ levels were determined to be −3.86, −3.83, and −3.82 eV for ITO/NiO_*x*_, ITO/NiO_*x*_/SR-1, and ITO/NiO_*x*_/SR-2, respectively. Further, the *E*_CB_ values of the substrates, summarized in Table 1, were estimated from the *E*_g_ and *E*_VB_. In addition, Kelvin probe force microscopy (KPFM) was performed to evaluate the work-function variations of the ITO/NiO_*x*_ films with and without SR molecules, as illustrated in Figure S13. The measured contact potential difference (CPD) distributions for ITO/NiO_*x*_, ITO/NiO_*x*_/SR-1, and ITO/NiO_*x*_/SR-2 were −177.46 mV, −163.90 mV, and −170.30 mV, respectively. The positive CPD shifts observed upon introduction of SR-1 (+13.6 mV) and SR-2 (+7.2 mV) indicate an upward adjustment of the surface work function. These results are consistent with the UPS data, further confirming effective interfacial modification by SR molecules.

Fourier-transform infrared (FT-IR) spectroscopy was employed to analyze the chemical interactions between the SR molecules and both NiO_*x*_ and PbI_2_. The FT-IR spectra of the ITO/NiO_*x*_/SR-1 and ITO/NiO_*x*_/SR-2 films exhibit broader and more intense peaks at 1614 cm^–1^ and 1621 cm^–1^, respectively, compared to the ITO/NiO_*x*_ film, with slight shifts to lower wavenumbers (Figure S14­(a)). These shifts suggest strong interfacial interactions between the imine groups of the phenanthroimidazole moiety and NiO_*x*_. The red shift is attributed to Lewis acid–base interactions, where the Ni centers in NiO_*x*_ coordinate with the electron-rich imine nitrogen, weakening the C=N bond and lowering its vibrational frequency.^53^ The peak broadening further indicates enhanced electronic coupling and charge transfer at the NiO_*x*_/SR interface, which is beneficial for improving interfacial charge transport. Similarly, the FT-IR spectra of pristine SR-1 and SR-2 exhibit characteristic C=N stretching vibrations at 1608 cm^–1^ and 1601 cm^–1^, respectively. Upon grinding with PbI_2_, these peaks shift slightly to 1603 cm^–1^ (SR-1) and 1600 cm^–1^ (SR-2), as shown in Figure S14­(b). This shift indicates that the lone-pair electrons on the imine nitrogen form intermolecular chemical interactions with the Pb^2+^ ions. Such interactions are expected to effectively reduce trap state density, enhance charge transport, and suppress charge recombination at the NiO_*x*_/perovskite interfaces.^51,53^

The XPS analyses were conducted to assess the electronic state changes of the nickel element from the redox reaction at the NiO_*x*_/SR-molecule interfaces (Figure 2). The deconvolution peaks of Ni 2p states at BEs of 852.32 and 854.12 eV are observed for the bare NiO_*x*_ film, 852.38 and 854.25 eV for the NiO_*x*_/SR-1 film, and 852.27 and 854.06 eV for the NiO_*x*_/SR-2 film. These peaks are assigned to the electronic states of Ni^2+^ and Ni^3+^ sites.^35^ The Ni^3+/^Ni^2+^ ratios in NiO_*x*_, NiO_*x*_/SR-1, and NiO_*x*_/SR-2 films were calculated, based on the integrated areas of these deconvolution peaks, to be 3.18, 3.51, and 3.30, respectively. The direct interaction of Ni^3+^ vacant sites and the perovskite layer on the NiO_*x*_ surface is prone to the degradation of the MAPbI_3_ structure through the redox reaction between iodide species and Ni^3+^ sites, where the Ni^3+^ ions act as Lewis acids.^31^ Ni^≥3+^ can oxidize iodide species and deprotonate cationic amines (CH_3_NH_3_^+^) by playing a dual role as a Lewis electron acceptor and a Bronsted proton acceptor.^24^ On the other hand, the presence of Ni^3+^ significantly improved the p-type conductivity hole mobility and simultaneously inhibited the perovskite degradation, which has been extensively established in recent research.^27,34,45^ It is necessary to reach an optimal Ni^3+^/Ni^2+^ ratio to simultaneously mitigate the unfavorable redox reaction at the NiO_*x*_/perovskite interface and promote the p-type characteristics of NiO_*x*_.^54^ The lone pair electrons on heteroatoms of SR molecules serve as Lewis base sites to compensate for unfavorable redox reactions between the NiO_*x*_ and MAPbI_3_ layer. The ratio of Ni^3+^/Ni^2+^ slightly increases for both NiO_*x*_/SR-1 and NiO_*x*_/SR-2 compared to that of bare NiO_*x*_. Hence, it is conceivable that the p-type characteristics of NiO_*x*_ films are enhanced through the incorporation of SR molecules.^55^

**Figure 2:**
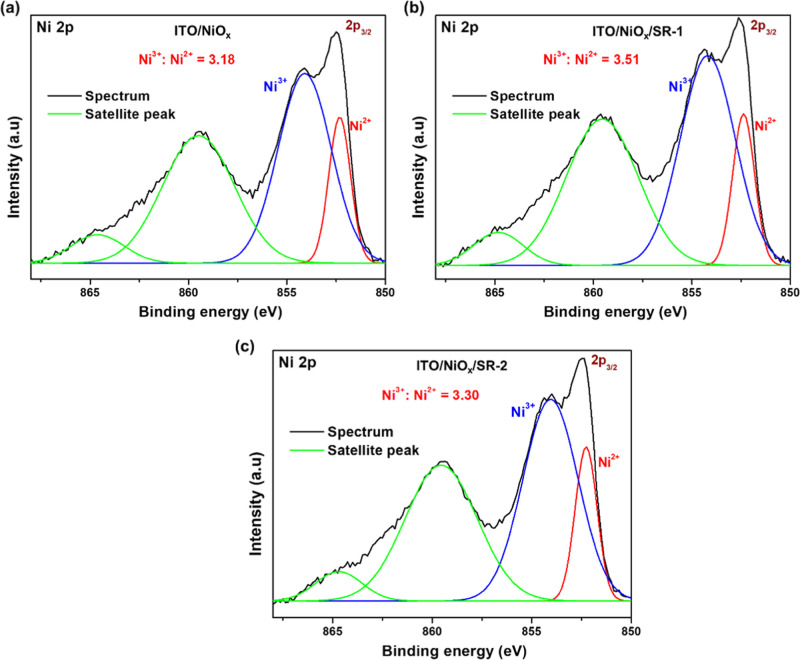
(a–c) The XPS spectra of the nickel element (Ni, 2p) for the substrates recorded for ITO/NiO_*x*_, ITO/NiO_*x*_/SR-1, and ITO/NiO_*x*_/SR-2.

XPS measurements were performed on a MAPbI_3_ layer deposited on NiO_*x*_, NiO_*x*_/SR-1, and NiO_*x*_/SR-2 films to probe the chemical environment of Pb and I elements in the perovskite. Figures 3a and 3b show the XPS spectra of the Pb 4f and I 3d electronic states, respectively. It is well established that one of the major factors contributing to the poor performance of PSCs is the undesired degradation of MAPbI_3_ to PbI_2_.^56^ The BE shift from a higher to a lower energy region in the SR-interlayered films suggests an increased electron density around Pb and I atoms, likely arising from strong interactions such as Pb–N coordination and hydrogen bonding with iodide ions (I^–^), leading to effective passivation of interfacial defects. These phenomena collectively enhance charge transport, reduce charge recombination, and improve the stability of MAPbI_3_ films.^57^ The observed results strongly support the role of SR-IFLs as effective passivation layers at the NiO_*x*_/perovskite interface.

**Figure 3:**
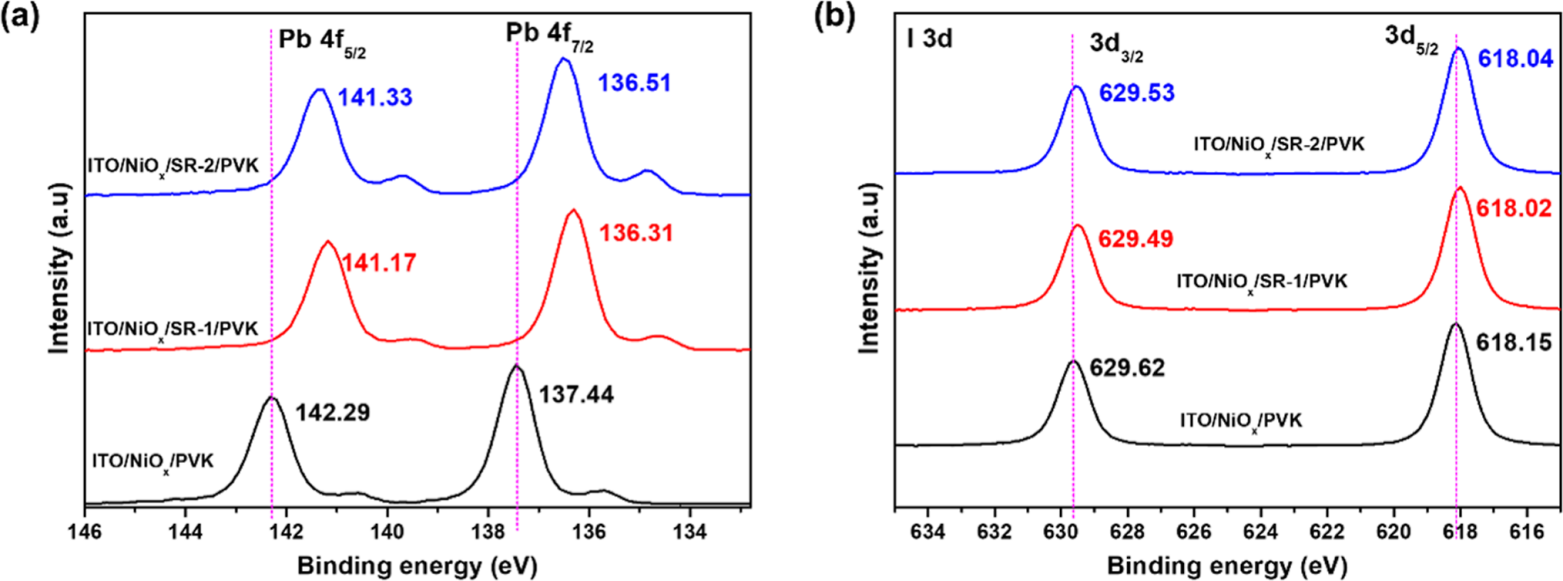
(a,b) XPS spectra of lead (Pb 4f) and iodine (I 3d) elements for the perovskite films spin-coated on the ITO/NiO_*x*_, ITO/NiO_*x*_/SR-1, and ITO/NiO_*x*_/SR-2 substrates.

The morphological characteristics of MAPbI_3_ films deposited on top of bare NiO_*x*_, NiO_*x*_/SR-1, and NiO_*x*_/SR-2 were characterized by field emission scanning electron microscopy (FE-SEM), as shown in Figure 4a,c. The surface morphology of the MAPbI_3_ film in the presence of SR-IFLs shows a uniform surface, and the grain sizes are larger than that of the control device without SR-IFL. The average grain sizes were found to be 0.81, 1.1, and 1.06 μm for the bare NiO_*x*_/perovskite, NiO_*x*_/SR-1/perovskite, and NiO_*x*_/SR-2/perovskite, respectively. These larger, more uniform grains indicate improved crystallization kinetics and reduced grain boundary density, which are beneficial for charge transport and device performance. As shown in Figure S15, the water contact angles are 19.8, 27.5, and 26.8° for bare NiO_*x*_, ITO/NiO_*x*_/SR-1, and ITO/NiO_*x*_/SR-2, respectively, indicative of a slight increase of NiO_*x*_ surface hydrophobicity after depositing a thin layer of SR molecules. The surface roughness of the MAPbI_3_ film in the presence of SR molecules was evaluated by using tapping-mode atomic force microscopy. As displayed in Figure S16, the average root-mean-square (*R*_a_) roughness decreased from 13.6 nm for the control to 11.6 and 12.0 nm for the SR-1- and SR-2-passivated MAPbI_3_ films, which is beneficial for perovskite contact with the top electron transport layer.

**Figure 4:**
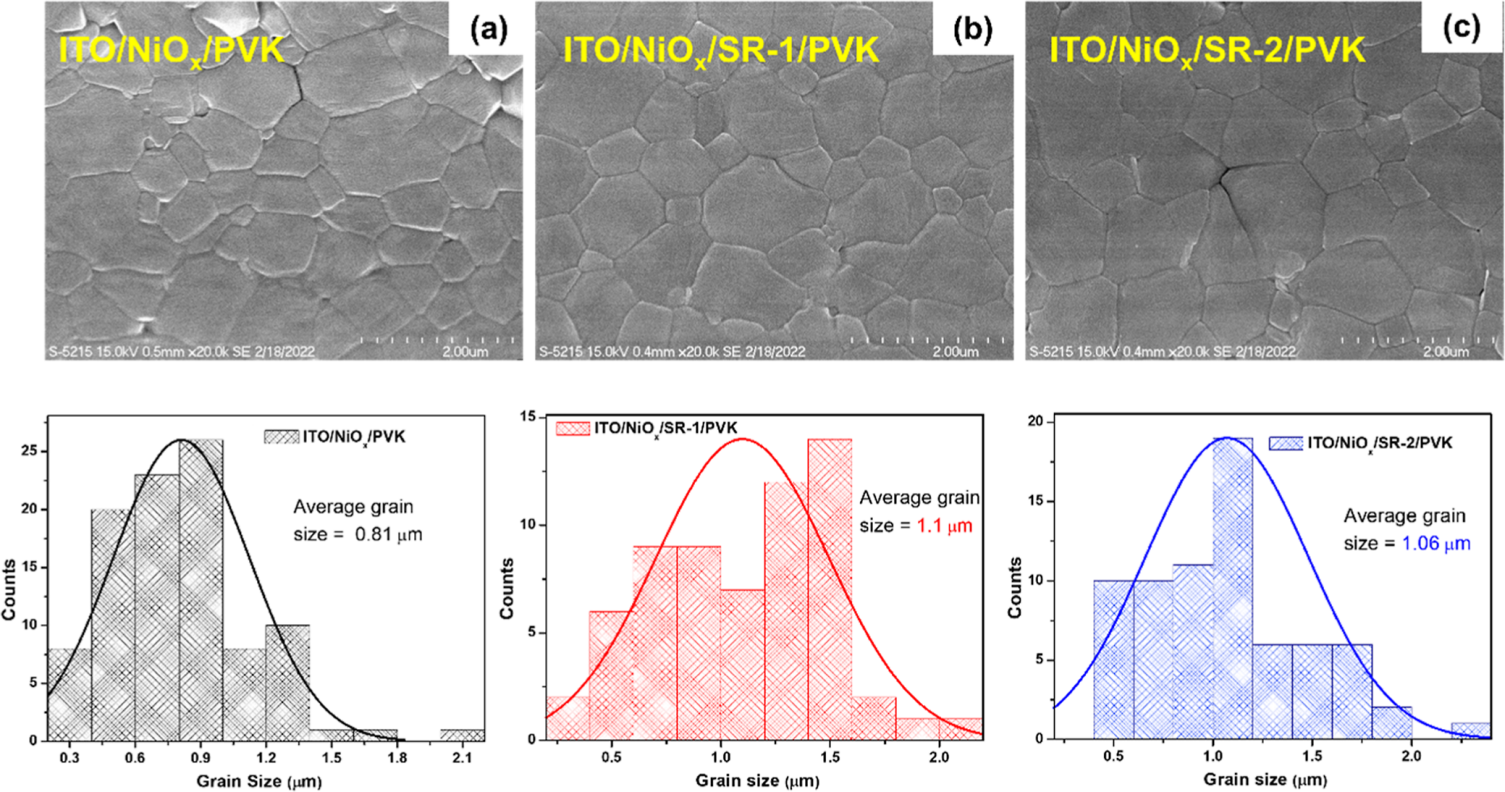
Top view FE-SEM images and their corresponding statistical histogram of grain size distributions. The scale bar is 2 mm. (a) Control, (b) ITO/NiO_*x*_/SR-1, and (c) ITO/NiO_*x*_/SR-2.

Cross-section SEM images, shown in Figure S17, were obtained for the device architectures (a) ITO/NiO_*x*_/MAPbI_3_, (b) ITO/NiO_*x*_/SR-1/MAPbI_3_, and (c) ITO/NiO_*x*_/SR-2/MAPbI_3_. The perovskite layers exhibited a uniform thickness and compact morphology with well-defined interfaces between each layer. The NiO_*x*_ layer, with or without SR IFL, exhibited a thickness of ∼20 nm. The ultrathin SR IFLs (∼5–10 nm) were not directly discernible in the SEM images due to resolution limits. Previous studies have shown that NiO_*x*_ layers with a thickness of 20–30 nm provide optimal hole extraction while minimizing series resistance.^58^ Thinner NiO_*x*_ films (<20 nm) lead to incomplete substrate coverage and shunting paths, whereas thicker films (>40 nm) increase series resistance and degrade device performance. Therefore, maintaining a NiO_*x*_ thickness in the range 20–30 nm offers a balanced condition that ensures the high performance of the fabricated devices.

Figure 5a displays the UV–vis absorption spectra of the perovskite films with and without SR molecules. All perovskite films exhibit identical absorption edges, suggesting that SR molecules do not alter the band gap of perovskite films. The conventional Tauc plot revealed a consistent *E*_g_ value of 1.6 eV for all perovskite films, as illustrated in Figure S18. The crystallinity of the MAPbI_3_ layer on top of the NiO_*x*_/SR layer was evaluated by powder X-ray diffraction (PXRD), as illustrated in Figure 5b. The same diffraction peaks appeared at 14.0° (110), 28.4° (220), and 31.8° (310) for NiO_*x*_/perovskite and NiO_*x*_/SR/perovskite films, suggesting that the perovskite maintains a consistent structural pattern when SR molecules are incorporated as an IFL at the interface between NiO_*x*_ and the perovskite. In comparison to the control device, the perovskite films with the SR interfacial layer exhibited higher crystallinity, as evidenced by the narrower full width at half-maximum (fwhm) of diffraction peaks than those of the control without SR (Table S4). The defects in the MAPbI_3_ surface were effectively passivated by the SR IFL, which leads to enhanced crystallinity of the perovskite film and significantly promotes the grain sizes during the nucleation of the perovskite (vide infra).^59^

**Figure 5:**
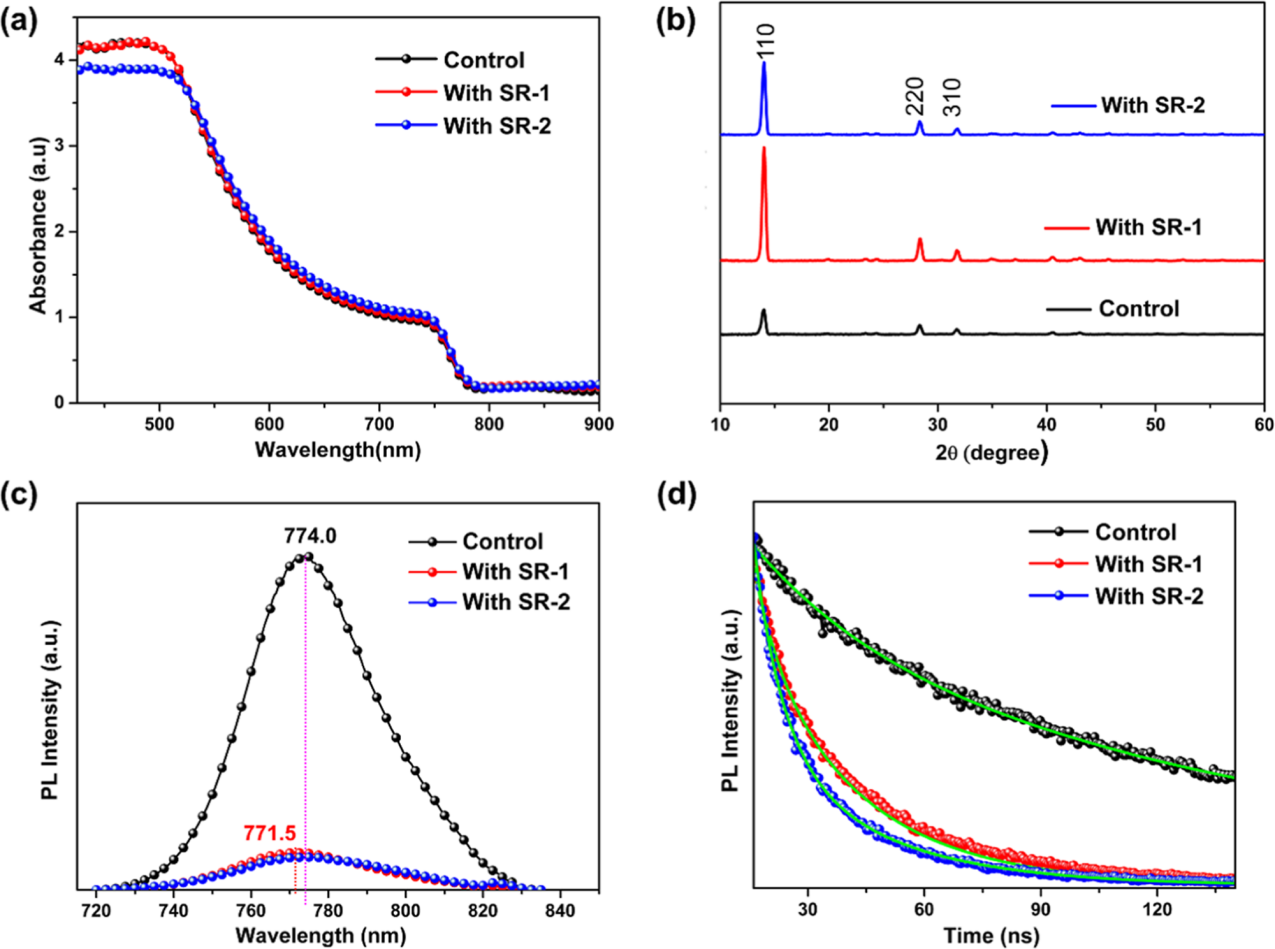
SR molecules are embedded at the NiO_*x*_ and perovskite interface. (a) UV–vis absorption spectra. (b) PXRD patterns. (c) Steady-state photoluminescence (PL) spectra. (d) Time-resolved photoluminescence (TRPL) decay curves.

The PL and TRPL measurements were carried out to assess the dynamics of charge transfer properties at the NiO_*x*_/perovskite interface in the presence of the SR-IFL. A significant PL quenching was observed for the NiO_*x*_/SR/perovskite films (Figure 5c). Both NiO_*x*_/SR/perovskite films showed significant PL quenching and blue shift in their PL maxima compared to that of the NiO_*x*_/perovskite film, indicative of the effective charge extraction and passivation effect with minimized interfacial defects in the NiO_*x*_/SR/perovskite interface.^60^ As expected, the NiO_*x*_/SR/perovskite films revealed faster PL decay dynamics than that of the control device, as illustrated in Figure 5d, and Table S5 further echoes the effective charge extraction at the interface between NiO_*x*_ and perovskite.

The space-charge-limited current (SCLC) measurements revealed the charge carrier mobilities and defect densities at the NiO_*x*_/SR/perovskite interface. The fitted values from SCLC measurements for trap-filled-limit voltage (*V*_TFL_), hole mobility (μ_h_), and trap density (*N*_*t*_) are summarized in Table S6. The corresponding current density–voltage (*J*–*V*) curves and their device architecture are depicted in Figure S19. The hole mobilities are determined to be 1.27 × 10^–3^, 1.61 × 10^–3^, and 1.49 × 10^–3^ cm^2^ V^–1^ s^–1^ for the control device and NiO_*x*_/SR-1- and NiO_*x*_/SR-2-derived devices, respectively. The hole mobility of the SR-passivated devices exhibited a notable enhancement compared to that of the control device. The trap densities, calculated from the trap-filled-limit voltages based on eq S3, are 3.81 × 10^15^, 1.86 × 10^15^, and 2.18 × 10^15^ cm^–3^ for the control, SR-1-based, and SR-2-based devices, respectively. As per the SCLC analysis, the interface properties between NiO_*x*_ and perovskite film showed improvement by incorporating an interfacial layer of SR molecules. Specifically, the improved hole mobilities and reduced trap densities with the SR-based interfacial layer are expected to result in effective charge extraction and minimized charge carrier recombination losses at the NiO_*x*_/perovskite interfaces.^26^

## 3 Photovoltaic Performance of PSCs

Figure 6a shows the *J*–*V* curves of champion devices measured under 1 sun (100 mW cm^–2^, AM 1.5G) illumination, and the relevant photovoltaic parameters are summarized in Table 2. A schematic view of the inverted device, fabricated with the configuration ITO/NiO_*x*_ (without or with IFLs)/perovskite/PCBM/BCP/Ag, is illustrated in Figure S20. The best PCE of 20.3% came from the SR-1-passivated device with a short-circuit current (*J*_sc_) of 23.5 mA cm^–2^, an open-circuit voltage (*V*_oc_) of 1.09 V, and a fill factor (FF) of 79.2%. The SR-2-passivated device produced a PCE of 19.9% with a *J*_sc_ of 23.2 mA cm^–2^, a *V*_oc_ of 1.06 V, and an FF of 80.8%. Both devices with SR-1 and SR-2 IFLs outperformed the pristine device without SR-IFLs (PCE of 17.4% with a *J*_sc_ of 21.4 mA cm^–2^, a *V*_oc_ of 1.09 V, and an FF of 74.4%). SR-2 contains an additional phenyl spacer relative to that of SR-1. Its slightly lower PCE is attributed to the reduced molecular planarity and less efficient packing at the NiO_*x*_/perovskite interface. The extra phenyl ring introduces greater torsional flexibility, which can hinder π–π-stacking and interfacial contact. Consequently, charge extraction becomes slightly less efficient than that in SR-1, despite SR-2 exhibiting a deeper work function. These findings account for the higher PCE and *V*_oc_ observed in SR-1-based devices and highlight the critical role of balancing energetic alignment with interfacial molecular packing for achieving superior photovoltaic performance. In addition, the SR-1-passivated device showed negligible *J*–*V* hysteresis behavior compared to the pristine device, as shown in Figure S21 and Table S7. To further examine the charge transport characteristics of NiO_*x*_ films with and without SR interlayers, conductivity measurements were conducted using devices with a FTO/NiO_*x*_/(with or without SR)/Ag configuration (Figure S22). The results reveal that both SR interlayers improved the electrical properties of NiO_*x*_ following the order NiO_*x*_/SR-1 > NiO_*x*_/SR-2 > pristine NiO_*x*_. This trend aligns well with the corresponding Ni^3+^/Ni^2+^ ratios of 3.18 (NiO_*x*_), 3.51 (SR-1), and 3.30 (SR-2). The higher proportion of Ni^3+^ increased the hole carrier density, which enhances p-type conductivity and facilitates more efficient hole extraction at the NiO_*x*_/perovskite interface.^27^ While SR-2 also enhances conductivity moderately, consistent with its intermediate device performance, the superior *J*_sc_ and fill factor of SR-1-based devices can be attributed to their higher conductivity and optimized interfacial charge transport.

**Figure 6:**
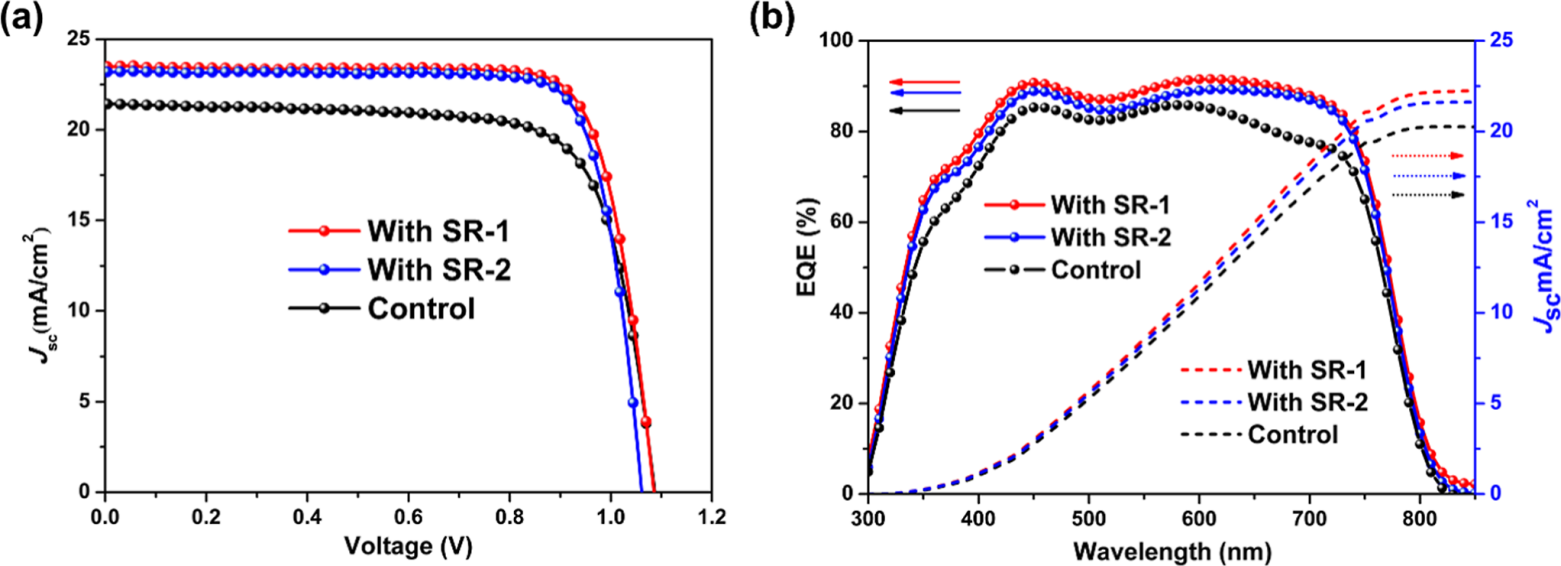
(a) *J*–*V* curves of NiO_*x*_-based p–i–n devices with and without SR-IFLs. (b) The EQE spectra (solid lines) and the integrated *J*_sc_ (dashed lines).

**Table 2:** Photovoltaic Parameters of SR-1- and SR-2-Passivated Devices^a^

devices	*V*_oc_ (V)	*J*_sc_ (mAcm^–2^)	FF (%)	PCE (%)
SR-1	1.07 ± 0.01 (1.09)	22.5 ± 0.5 (23.5)	78.5 ± 1.5 (79.2)	19.1 ± 0.7 (20.3)
SR-2	1.07 ± 0.01 (1.06)	22.3 ± 0.5 (23.2)	78.9 ± 0.9 (80.8)	18.7 ± 0.5 (19.9)
control	1.05 ± 0.02 (1.09)	21.2 ± 0.7 (21.4)	74.8 ± 2.0 (74.4)	16.7 ± 0.6 (17.4)

a The average is obtained based on 20 devices for SR-1 and control and 16 devices for SR-2. The data of the champion devices are shown in parentheses.

Figure 6b shows the external quantum efficiency (EQE) spectra. The integrated photocurrent densities over the whole spectral region are well-matched with the *J*_sc_ values obtained from the *J*–*V* curves. The enhanced EQE response observed in the 600–800 nm region can be attributed to the improved film morphology and crystallinity of the perovskite layer induced by the SR-1 and SR-2 interlayers. As shown in the SEM images (Figure 4), the perovskite films grown on SR-modified NiO_*x*_ surfaces exhibited larger grain sizes and more compact coverage compared to those of the control. Consistently, XRD patterns (Figure 5b) revealed an enhanced diffraction peak intensity with reduced fwhm, confirming improved crystallinity. These morphological improvements suppress trap-assisted recombination and facilitate more efficient charge collection, thereby leading to the enhanced EQE response. Incorporating SR-IFLs into the NiO_*x*_/perovskite interface resulted in improved crystallinity and increased grain size of the perovskite film, which subsequently enhanced the hole mobility and decreased the interface trap density. The increased Ni^3+^/Ni^2+^ ratio of the NiO_*x*_ layer upon depositing the SR-IFLs leads to improved p-type characteristics of NiO_*x*_. As a result, an improvement in device performance is observed in p–i–n PSCs with the addition of the SR-IFLs. The reproducibility of the device performance is validated by the statistic distributions of photovoltaic parameters and PCEs shown in Figure S23.

The steady-state photocurrent densities and PCE values at the maximum power point tracking were recorded to assess the reliability of the device outputs. As depicted in Figure S24, both SR-1- and SR-2-passivated devices produced stabilized maximum photocurrent densities and PCEs. Meanwhile, the charge recombination kinetics at the NiO_*x*_/perovskite interface were accessed using electrochemical impedance spectra (EIS) *J*–*V* measurements in dark conditions. Figure S25a shows the Nyquist plots of the control and SR-passivated devices, and the results are summarized in Table S8. The estimated charge recombination resistance (*R*_rec_) values for the control, SR-1, and SR-2 devices are 428, 743, and 723 Ω, respectively. The higher *R*_rec_ values in devices comprising SR molecules imply reduced charge recombination than the control device, consistent with the improved FF and *V*_oc_ listed in Table 2.^61^ The dark *J*–*V* curves (Figure S25b) revealed that the leakage current follows the order NiO_*x*_/SR-1 < NiO_*x*_/SR-2 < NiO_*x*_, confirming that both SR-1 and SR-2 reduce leakage compared to the control device. The lowest current leakage observed for NiO_x/_SR-1 indicates a more effective passivation of interfacial trap states, leading to suppressed carrier recombination. These results demonstrate that the incorporation of SR interlayers improves interfacial quality, which correlates well with the enhanced photovoltaic performance of the corresponding devices.

The long-term thermal stability of PSC devices incorporating SR-IFLs was evaluated following the ISOS-85 protocol.^62^ Thermal stability tests were conducted under an inert atmosphere under unencapsulated conditions, with continuous heating at 60 °C and a relative humidity of 50–60%, as illustrated in Figure 7. Under these conditions, the control device exhibited a significant decline in PCE, dropping below 20% of its initial value after 350 h. This degradation is primarily attributed to the thermal decomposition of MAPbI_3_ into PbI_2_. Additionally, the migration of iodide (I^–^) and iodine species (I_2_) toward the silver (Ag) electrode surface contributes to device instability. A comparative analysis of the thermal stability of NiO_*x*_-based inverted PSCs from the literature is summarized in Table S9.^63–65^ The PCEs of SR-passivated PSCs were retained at over 94% after 350 h. The operational stability of the control, SR-1, and SR-2 devices was evaluated under continuous light soaking (AM1.5G) with maximum power point tracking (MPPT) at room temperature and 85–90% relative humidity (Figure S26). The control device showed rapid degradation, retaining only ∼69% of its initial PCE. In contrast, devices incorporating SR interlayers exhibited markedly improved stability: SR-1 retained ∼89% of its initial PCE and ∼88% of *J*_sc_, while SR-2 achieved the best stability, maintaining ∼96% of both parameters. The enhanced stability is attributed to the SR molecules’ effective passivation of interfacial defects and their strong chemical stability, which suppress charge recombination and prevent interfacial deterioration. These results highlight a trade-off between performance and stability; SR-1 provides higher photocurrent generation, whereas SR-2 offers enhanced long-term stability. Overall, the excellent passivation capability of PIM-based OSMs makes them promising contenders for achieving high device performance and long-term stability in inverted p–i–n PSCs.

**Figure 7:**
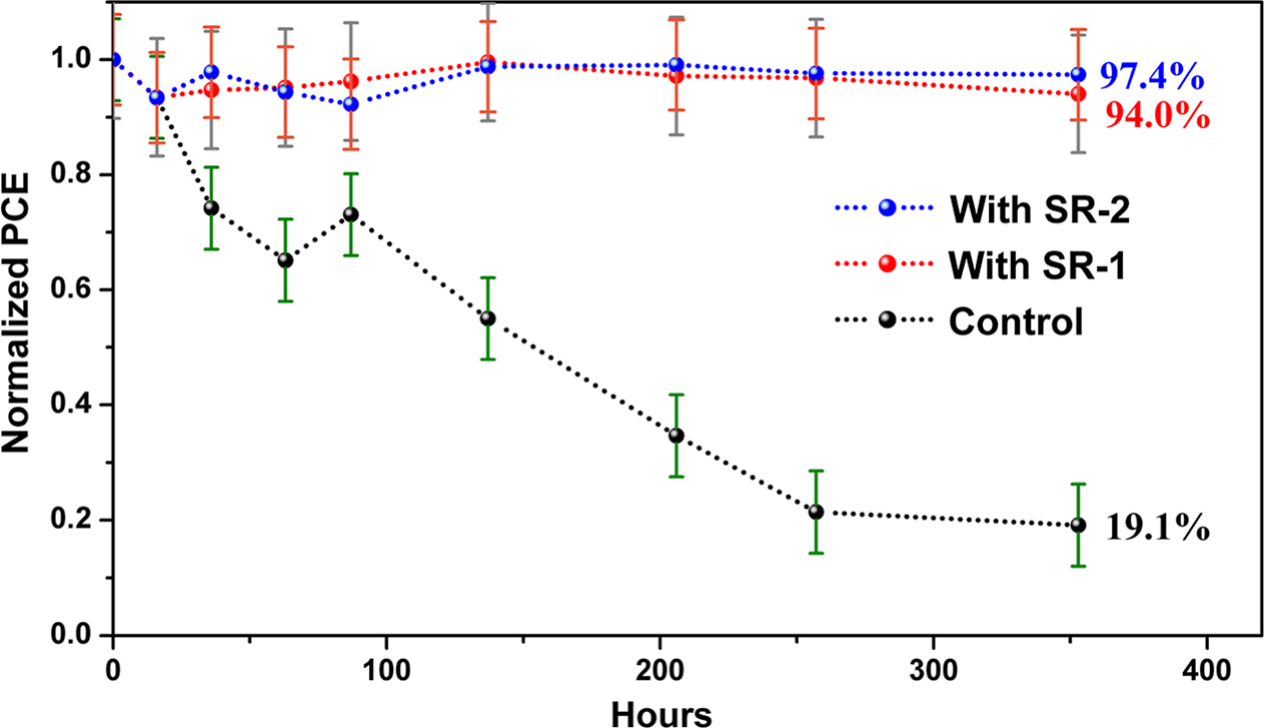
Validated thermal aging stability of unencapsulated NiO_*x*_-based PSCs with and without SR molecules under ambient air with relative humidity at 50–60%. The error bars are based on measurements of 3 devices.

## 4 Conclusion

In summary, we successfully synthesized two PIM-containing OSMs, SR-1 and SR-2, using straightforward synthetic methods. We have demonstrated here that the SR-based OSMs are promising materials for an interfacial passivation layer. SR molecules were utilized as ultrathin IFLs at the NiO_*x*_/perovskite interface without using any dopants in NiO_*x*_ for inverted PSCs. The Ni^3+^/Ni^2+^ ratio was increased, and the defects in the bottom surface of the MAPbI_3_ film were passivated after depositing a thin layer of SR-1 or SR-2 molecules on top of the NiO_*x*_ layer. A systematic analysis of the redox characteristics of these SR molecules at the NiO_*x*_/perovskite interface was conducted to reveal that the SR-based devices improved the hole extraction and the crystallinity of the perovskite film with smooth interfaces and minimized the defect density. As a result, the photovoltaic device with a configuration of ITO/NiO_*x*_/SR-1/MAPbI_3_/PCBM/BCP/Ag utilizing SR-1 as a passivation layer achieved an excellent PCE of 20.3% compared to that of the control device (17.4%). The 20.3% PCE represents one of the best device performances reported for MAPbI_3_-based p–i–n devices in recent years (see Table S10). The incorporation of SR-IFLs resulted in the high recorded PCE while demonstrating improved long-term stability. The device retained over >94% of its initial performance after 350 h under ISOS-85 testing conditions, highlighting the effectiveness of SR-IFLs in enhancing the operational durability of inverted perovskite solar cells under thermal stress. The performance of the device based on SR-1 is one of the highest PCEs among those reported in the literature for the OSM of imidazole derivatives used as either HTM or IFL in the MAPbI_3_-based PSCs. The facile synthesis and excellent photovoltaic performance make them viable as IFL materials for emerging, efficient, dopant-free inverted PSCs.

## 5 Experimental Section

### 5.1 Materials and Synthesis

Scheme 1 outlines the synthetic procedures for preparing SR-1 and SR-2 molecules. Compound 1 and *p*-OMeTPA were prepared according to the literature.^66,67^ All other chemicals were commercially available and used without further purification.

### 5.2 Synthesis of SR-1

To a 100 mL one-necked round-bottom flask, a mixture of compound 1 (300 mg, 0.49 mmol), *para*-methoxydiphenylamine (*p*-OMeDPA) (362 mg, 1.57 mmol), [(^*t*^Bu)_3_PH]­BF_4_ (22 mg, 0.074 mmol), Pd_2_(dba)_3_ (68 mg, 0.074 mmol), and sodium *tert*-butoxide (89 mg, 0.98 mmol) was dissolved in degassed toluene. The mixture was refluxed at 110 °C under a N_2_ atmosphere for 48 h. After cooling, the obtained suspension was diluted with DCM and washed four times with brine. The organic phase was collected and dried over MgSO_4_. After solvent evaporation, the residue was subjected to column chromatography on silica gel using hexane/DCM (1:1, v/v) as the eluent to obtain a yellow solid of SR-1 (66% yield). ^1^H NMR (400 MHz, CDCl_3_): δ (ppm) 8.36–8.31 (m, 3H), 7.22–7.20 (m, 5H), 7.16 (d, *J* = 6.87 Hz, 1H), 7.15–7.10 (m, 6H), 7.08 (d, *J* = 2.34 Hz, 1H), 7.0–6.96 (m, 4H), 6.91–6.86 (m, 4H), 6.85–6.83 (m, 4H), 6.80–6.76 (m, 4H), 6.75–6.720 (m, 4H), 6.67 (d, *J* = 8.82 Hz, 2H), 6.65 (d, *J* = 2.26 Hz, 1H), 3.83 (s, 6H), 3.80 (s, 6H), 3.77 (s, 6H). ^13^C NMR (100 MHz, CDCl_3_): δ (ppm): 156.1, 155.6, 155.6, 155.5, 150.8, 148.9, 146.9, 146.3, 141.6, 141.1, 140.3, 138.4, 137.2, 129.8, 129.7, 129.1, 128.6, 128.1, 127.3, 127.0, 126.4, 126.1, 124.1, 123.5, 123.2, 123.1, 122.9, 121.8, 121.2, 119.2, 118.8, 114.72, 114.68, 114.66, 113.6, 111.6, 55.53, 55.50, 55.46. HRMS (MALDI): *m*/*z* calcd for C_69_H_57_N_5_O_6_: 1051.4309 [M]^+^; found, 1051.4338.

### 5.3 Synthesis of SR-2

In a 20 mL pressure tube, a mixture of compound 1 (100 mg, 0.165 mmol), *para*-methoxytriphenylamine (*p*-OMeTPA) (227 mg, 0.528 mmol), and Pd­(PPh_3_)_4_ (0.0247 mmol) was dissolved in toluene (10 mL), followed by addition of 1 mL of a 2 M K_2_CO_3_ aqueous solution. The reaction mixture was stirred at 110 °C under a N_2_ atmosphere for 2 days. After cooling, the suspension was diluted with DCM and washed by brine four times. The organic phase was combined and dried over MgSO_4_. After the solvent was removed, the obtained residue was purified by column chromatography on silica gel using hexane/DCM (1:1, v/v) as an eluent to give a yellow solid of SR-2 (55% yield). ^1^H NMR (400 MHz, acetone-*d*_6_/CDCl_3_ (3:1 v/v)): δ (ppm) 8.95 (d, J = 1.55, 1H), 8.55 (d, *J* = 8.6 Hz, 2H), 7.91 (s, 1H), 7.73 (d, *J* = 7.37 Hz, 1H), 7.67 (d, *J* = 8.63 Hz, 2H), 7.63–7.58 (m, 6H), 7.53 (d, *J* = 6.54 Hz, 2H), 7.42–7.37 (m, 5H), 7.06 (d, *J* = 8.84 Hz, 6H), 7.01–6.97 (m, 9H), 6.87 (d, *J* = 2.01 Hz, 2H), 6.86–6.83 (m, 12H), 6.68 (d, *J* = 8.65 Hz, 2H), 3.77 (s, 12H), 3.75 (s, 6H). ^13^C NMR (100 MHz, acetone-*d*_6_/CDCl_3_ (3:1 v/v)): δ (ppm) 157.1, 157.0, 157.0, 151.0, 149.4, 149.2, 149.1, 141.5, 141.4, 141.3, 141.2, 140.0, 139.6, 138.3, 138.1, 133.2, 132.1, 132.0, 131.1, 130.6, 130.3, 130.1, 129.4, 128.4, 128.3, 128.1, 127.9, 127.7, 127.69, 127.53, 127.48, 127.4, 126.3, 125.3, 124.6, 123.9, 123.4, 121.2, 120.7, 120.0, 118.3, 115.5, 55.5. HRMS (MALDI): *m*/*z* calcd for C_87_H_69_N_5_O_6_: 1279.5248 [M]^+^; found, 1279.5035.

### 5.4 Device Fabrication and Performance Measurement

The detailed device fabrication procedures and performance measurements are included in the Supporting Information.

## Supplementary Material



## Notes

Experimental methods and characterization data including NMR spectra and high-resolution mass spectrometry data, detailed device fabrication procedures, and additional physical characterization data for materials and devices (PDF)

## References

[1] Gong C., Li H., Xu Z., Li Y., Wang H., Zhuang Q., Wang A., Li Z., Guo Z., Zhang C. (2024). Efficient and stable inverted perovskite solar cells enabled by homogenized
PCBM with enhanced electron transport. Nat.
Commun..

[2] Wang K., Xu Z., Li K., Li R., Guo Z., Yang Y., Huang J., Mohammed O. F., Zang Z. (2025). Amidino-based ligand
enables oriented crystallization and boosted carrier extraction for
inverted perovskite solar cells and modules. Joule.

[3] Bati A. S., Zhong Y. L., Burn P. L., Nazeeruddin M. K., Shaw P. E., Batmunkh M. (2023). Next-generation applications
for
integrated perovskite solar cells. Commun. Mater..

[4] Stranks S. D., Eperon G. E., Grancini G., Menelaou C., Alcocer M. J., Leijtens T., Herz L. M., Petrozza A., Snaith H. J. (2013). Electron-hole
diffusion lengths exceeding 1 micrometer in an organometal trihalide
perovskite absorber. Sci..

[5] Herz L. M. (2017). Charge-carrier
mobilities in metal halide perovskites: fundamental mechanisms and
limits. ACS Energy Lett..

[6] Kojima A., Teshima K., Shirai Y., Miyasaka T. (2009). Organometal
halide
perovskites as visible-light sensitizers for photovoltaic cells. J. Am. Chem. Soc..

[7] Park J., Kim J., Yun H.-S., Paik M. J., Noh E., Mun H. J., Kim M. G., Shin T. J., Seok S. I. (2023). Controlled growth
of perovskite layers with volatile alkylammonium chlorides. Nat..

[8] Lin Y.-S., Abate S. Y., Lai K.-W., Chu C.-W., Lin Y.-D., Tao Y.-T., Sun S.-S. (2018). New helicene-type
hole-transporting
molecules for high-performance and durable perovskite solar cells. ACS Appl. Mater. Interfaces.

[9] Lin Y.-D., Abate S. Y., Chung H.-C., Liau K.-L., Tao Y.-T., Chow T. J., Sun S.-S. (2019). Donor–acceptor–donor
type cyclopenta [2, 1-b; 3, 4-b′] dithiophene derivatives as
a new class of hole transporting materials for highly efficient and
stable perovskite solar cells. ACS Appl. Energy
Mater..

[10] Lin Y.-D., Lee K.-M., Chang S. H., Tsai T.-Y., Chung H.-C., Chou C.-C., Chen H.-Y., Chow T. J., Sun S.-S. (2021). Molecularly
engineered cyclopenta­[2,1-b;3,4-b′]­dithiophene-based hole-transporting
materials for high-performance perovskite solar cells with efficiency
over 19%. ACS Appl. Energy Mater..

[11] National Renewable Energy Laboratory (NREL) https://www.nrel.gov/pv/cell-efficiency.html (accessed November 12th 2025).

[12] Sha W. E., Ren X., Chen L., Choy W. C. (2015). The efficiency limit of CH_3_NH_3_PbI_3_ perovskite solar cells. Appl. Phys. Lett..

[13] Zhang S., Ye F., Wang X., Chen R., Zhang H., Zhan L., Jiang X., Li Y., Ji X., Liu S. (2023). Minimizing buried interfacial
defects for efficient inverted perovskite
solar cells. Sci..

[14] Yan P., Yang D., Wang H., Yang S., Ge Z. (2022). Recent advances
in dopant-free organic hole-transporting materials for efficient,
stable and low-cost perovskite solar cells. Energy Environ. Sci..

[15] Wang Y., Chen W., Wang L., Tu B., Chen T., Liu B., Yang K., Koh C. W., Zhang X., Sun H. (2019). Dopant-free small-molecule
hole-transporting material for inverted
perovskite solar cells with efficiency exceeding 21%. Adv. Mater..

[16] Wang M., Wang H., Li W., Hu X., Sun K., Zang Z. (2019). Defect passivation using ultrathin
PTAA layers for efficient and
stable perovskite solar cells with a high fill factor and eliminated
hysteresis. J. Mater. Chem. A.

[17] Wang C., Su Z., Chen L., Zhang H., Hui W., Liang D., Zheng G., Zhang L., Tang Z., Wen W. (2022). MoO_3_ doped PTAA for high-performance inverted perovskite
solar cells. Appl. Surf. Sci..

[18] Webb T., Sweeney S. J., Zhang W. (2021). Device architecture
engineering:
progress toward next generation perovskite solar cells. Adv. Funct. Mater..

[19] Zhu R., Guan N., Wang D., Bao Y., Wu Z., Song L. (2023). Review of defect passivation for
NiO_x_-based inverted perovskite
solar cells. ACS Appl. Mater. Interfaces.

[20] You J., Meng L., Song T.-B., Guo T.-F., Yang Y., Chang W.-H., Hong Z., Chen H., Zhou H., Chen Q. (2016). Improved air stability of perovskite solar cells via
solution-processed metal oxide transport layers. Nat. Nanotechnol..

[21] Zhang H., Zhao C., Yao J., Choy W. C. (2023). Dopant-free NiO_x_ nanocrystals: a low-cost
and stable hole transport material
for commercializing perovskite optoelectronics. Angew. Chem., Int. Ed..

[22] Hsiao K.-C., Lee B.-T., Jao M.-H., Lin T.-H., Hou C.-H., Shyue J.-J., Wu M.-C., Su W.-F. (2021). Chloride gradient
render carrier extraction of hole transport layer for high V_oc_ and efficient inverted organometal halide perovskite solar cell. Chem. Eng. J..

[23] Shin S. S., Lee S. J., Seok S. I. (2019). Metal Oxide
Charge Transport Layers
for Efficient and Stable Perovskite Solar Cells. Adv. Funct. Mater..

[24] Boyd C. C., Shallcross R. C., Moot T., Kerner R., Bertoluzzi L., Onno A., Kavadiya S., Chosy C., Wolf E. J., Werner J. (2020). Overcoming redox reactions at perovskite-nickel oxide
interfaces to boost voltages in perovskite solar cells. Joule.

[25] Traore B., Pedesseau L., Blancon J.-C., Tretiak S., Mohite A. D., Even J., Katan C., Kepenekian M. (2020). Importance
of vacancies and doping in the hole-transporting nickel oxide interface
with halide perovskites. ACS Appl. Mater. Interfaces.

[26] Yu S., Xiong Z., Zhou H., Zhang Q., Wang Z., Ma F., Qu Z., Zhao Y., Chu X., Zhang X. (2023). Homogenized NiO_x_ nanoparticles for improved hole transport
in inverted perovskite solar cells. Sci..

[27] Mann D. S., Kwon S. N., Thakur S., Patil P., Jeong K. U., Na S. I. (2024). Suppressing Redox Reactions at the
Perovskite-Nickel Oxide Interface
with Zinc Nitride to Improve the Performance of Perovskite Solar Cells. Small.

[28] Feng M., Wang Y., Liu F., Ren M., Wang H., Guo J., Chen Y., Miao Y., Zhao Y. (2024). Rational Buried Interface
Engineering of Inorganic NiO_x_ Layer toward Efficient Inverted
Perovskite Solar Cells. Sol. RRL.

[29] Liu Y., Ding B., Zhang G., Ma X., Wang Y., Zhang X., Zeng L., Nazeeruddin M. K., Yang G., Chen B. (2024). Synergistic Redox Modulation for
High-Performance Nickel Oxide-Based Inverted Perovskite Solar Modules. Adv. Sci..

[30] Li C., Zhang Y., Zhang X., Zhang P., Yang X., Chen H. (2023). Efficient Inverted
Perovskite Solar Cells with a Fill Factor Over
86% via Surface Modification of the Nickel Oxide Hole Contact. Adv. Funct. Mater..

[31] Mann D. S., Kwon S.-N., Patil P., Na S.-I. (2023). Revivification of
nickel oxide-perovskite interfaces via nickel nitrate to boost performance
in perovskite solar cells. Nano Energy.

[32] Kim J. H., Liang P. W., Williams S. T., Cho N., Chueh C. C., Glaz M. S., Ginger D. S., Jen A. K. Y. (2015). High-performance
and environmentally stable planar heterojunction perovskite solar
cells based on a solution-processed copper-doped nickel oxide hole-transporting
layer. Adv. Mater..

[33] Chen W., Zhou Y., Chen G., Wu Y., Tu B., Liu F. Z., Huang L., Ng A. M. C., Djurišić A. B., He Z. (2019). Alkali chlorides for the suppression of the interfacial recombination
in inverted planar perovskite solar cells. Adv.
Energy Mater..

[34] Zhou Y., Huang X., Zhang J., Zhang L., Wu H., Zhou Y., Wang Y., Wang Y., Fu W., Chen H. (2024). Interfacial Modification of NiO_x_ for Highly Efficient
and Stable Inverted Perovskite Solar Cells. Adv. Energy Mater..

[35] Ko S., Yong T., Kim S.-K., Park J. Y., Lee G., You H. R., Han S., Lee D. H., Choi S., Choi Y. C. (2023). A Top-Down
Strategy for Reforming the Characteristics
of NiO Hole Transport Layer in Inverted Perovskite Solar Cells. Sol. RRL.

[36] Li Z., Sun X., Zheng X., Li B., Gao D., Zhang S., Wu X., Li S., Gong J., Luther J. M. (2023). Stabilized
hole-selective layer for high-performance inverted pin perovskite
solar cells. Sci..

[37] Wang T., Cheng Z., Zhou Y., Liu H., Shen W. (2019). Highly efficient
and stable perovskite solar cells via bilateral passivation layers. J. Mater. Chem. A.

[38] Li Z., Jo B. H., Hwang S. J., Kim T. H., Somasundaram S., Kamaraj E., Bang J., Ahn T. K., Park S., Park H. J. (2019). Bifacial passivation
of organic hole transport interlayer
for NiO_x_-based p-i-n perovskite solar cells. Adv. Sci..

[39] Chen Y. C., Li Y. H., Chung C. L., Hsu H. L., Chen C. P. (2020). Triphenylamine
dibenzofulvene–derived dopant-free hole transporting layer
induces micrometer-sized perovskite grains for highly efficient near
20% for p-i-n perovskite solar cells. Prog.
Photovolt.: Res. Appl..

[40] Maddala S., Chung C.-L., Wang S.-Y., Kollimalayan K., Hsu H.-L., Venkatakrishnan P., Chen C.-P., Chang Y. J. (2020). Forming
a metal-free oxidatively coupled agent, bicarbazole, as a defect passivation
for htm and an interfacial layer in a p–i–n perovskite
solar cell exhibits nearly 20% efficiency. Chem.
Mater..

[41] Cheng H., Li Y., Zhao G., Zhao K., Wang Z.-S. (2019). Pyridine-terminated
conjugated organic molecules as an interfacial hole transfer bridge
for NiO_x_-based perovskite solar cells. ACS Appl. Mater. Interfaces.

[42] Lee C. C., Chen C. I., Fang C. T., Huang P. Y., Wu Y. T., Chueh C. C. (2019). Improving
performance of perovskite solar cells using
[7] helicenes with stable partial biradical characters as the hole-extraction
layers. Adv. Funct. Mater..

[43] Chen Y.-C., Lin D.-Z., Wang J.-C., Ni J.-S., Yu Y.-Y., Chen C.-P. (2021). Facile star-shaped tetraphenylethylene-based molecules
with fused ring-terminated diarylamine as interfacial hole transporting
materials for inverted perovskite solar cells. Mater. Chem. Front..

[44] Ramanujam R., Hsu H. L., Shi Z. E., Lung C. Y., Lee C. H., Wubie G. Z., Chen C. P., Sun S. S. (2024). Interfacial Layer
Materials with a Truxene Core for Dopant-Free NiO_x_-Based
Inverted Perovskite Solar Cells. Small.

[45] Li C., Wang X., Bi E., Jiang F., Park S. M., Li Y., Chen L., Wang Z., Zeng L., Chen H. (2023). Rational design of Lewis base molecules for stable and efficient
inverted perovskite solar cells. Sci..

[46] Chen W. C., Zhu Z. L., Lee C. S. (2018). Organic light-emitting
diodes based
on imidazole semiconductors. Adv. Opt. Mater..

[47] Sun P., Liu D., Zhu F., Yan D. (2023). An efficient solid-solution crystalline
organic light-emitting diode with deep-blue emission. Nat. Photonics.

[48] Sonmezoglu S., Akin S. (2020). Suppression of the
interface-dependent non-radiative recombination
by using 2-methylbenzimidazole as interlayer for highly efficient
and stable perovskite solar cells. Nano Energy.

[49] Feng J.-Y., Lai K.-W., Shiue Y.-S., Singh A., Kumar C. P., Li C.-T., Wu W.-T., Lin J. T., Chu C.-W., Chang C.-C. (2019). Cost-effective dopant-free
star-shaped oligo-aryl
amines for high performance perovskite solar cells. J. Mater. Chem. A.

[50] Akula S. B., Su C., Tingare Y. S., Lan H.-C., Lin Y.-J., Wang Y.-T., Jheng Y.-C., Lin X.-C., Chang Y.-C., Li W.-R. (2020). Thieno-imidazole
based small molecule hole transport materials for dopant-free, efficient
inverted (p–i–n) perovskite solar cells. J. Mater. Chem. C.

[51] Cheng Y., Fu Q., Zong X., Dong Y., Zhang W., Wu Q., Liang M., Sun Z., Liu Y., Xue S. (2021). Coplanar phenanthro­[9,10-d]­imidazole
based hole-transporting material enabling over 19%/21% efficiency
in inverted/regular perovskite solar cells. J. Chem. Eng..

[52] Nakka L., Cheng Y., Aberle A. G., Lin F. (2022). Analytical review of
spiro-OMeTAD hole transport materials: paths toward stable and efficient
perovskite solar cells. Adv. Energy Sustainability
Res..

[53] Zhou H., Yang L., Duan Y., Wu M., Li Y., Xu D., Zou H., Wang J., Yang S., Liu Z. (2023). 24.96%-Efficiency
FACsPbI_3_ Perovskite Solar Cells Enabled by an Asymmetric
1,3-Thiazole-2,4-Diammonium. Adv. Energy Mater..

[54] Hu T., Hou H., Peng J., Wu Q., He J., Yu H., Liu R., Hou T., Zhou X., Zhang M. (2023). 4-tert-butylpyridine
induced Ni^3+^/Ni^2+^ ratio modulation in NiO_x_ hole transport layer towards efficient and stable inverted
perovskite solar cells. Mater. Today Energy.

[55] Parida B., Yoon S., Ryu J., Hayase S., Jeong S. M., Kang D.-W. (2020). Boosting the Conversion Efficiency
Over 20% in MAPbI_3_ Perovskite Planar Solar Cells by Employing
a Solution-Processed
Aluminum-Doped Nickel Oxide Hole Collector. ACS Appl. Mater. Interfaces.

[56] Chen J., Park N.-G. (2020). Materials and Methods for Interface Engineering toward
Stable and Efficient Perovskite Solar Cells. ACS Energy Lett..

[57] Noel N. K., Abate A., Stranks S. D., Parrott E. S., Burlakov V. M., Goriely A., Snaith H. J. (2014). Enhanced
photoluminescence and solar
cell performance via Lewis base passivation of organic–inorganic
lead halide perovskites. ACS Nano.

[58] Lin Y.-R., Liao Y.-S., Hsiao H.-T., Chen C.-P. (2020). Two-step annealing
of NiO_x_ enhances the NiO_x_–perovskite
interface for high-performance ambient-stable p–i–n
perovskite solar cells. Appl. Surf. Sci..

[59] Xia X., Jiang Y., Wan Q., Wang X., Wang L., Li F. (2018). Lithium and Silver Co-Doped Nickel Oxide Hole-Transporting Layer
Boosting the Efficiency and Stability of Inverted Planar Perovskite
Solar Cells. ACS Appl. Mater. Interfaces.

[60] Shao Y., Xiao Z., Bi C., Yuan Y., Huang J. (2014). Origin and
elimination of photocurrent hysteresis by fullerene passivation in
CH_3_NH_3_PbI_3_ planar heterojunction
solar cells. Nat. Commun..

[61] Ghahremanirad E., Almora O., Suresh S., Drew A. A., Chowdhury T. H., Uhl A. R. (2023). Beyond protocols: Understanding the electrical behavior
of perovskite solar cells by impedance spectroscopy. Adv. Energy Mater..

[62] Khenkin M. V., Katz E. A., Abate A., Bardizza G., Berry J. J., Brabec C., Brunetti F., Bulović V., Burlingame Q., Di Carlo A. (2020). Consensus
statement
for stability assessment and reporting for perovskite photovoltaics
based on ISOS procedures. Nat. Energy.

[63] Wang Y., Ju H., Mahmoudi T., Liu C., Zhang C., Wu S., Yang Y., Wang Z., Hu J., Cao Y. (2021). Cation-size mismatch and interface stabilization
for efficient NiO_x_-based inverted perovskite solar cells
with 21.9% efficiency. Nano Energy.

[64] Wu T., Ono L. K., Yoshioka R., Ding C., Zhang C., Mariotti S., Zhang J., Mitrofanov K., Liu X., Segawa H. (2022). Elimination of light-induced degradation at
the nickel oxide-perovskite heterojunction by aprotic sulfonium layers
towards long-term operationally stable inverted perovskite solar cells. Energy Environ. Sci..

[65] Yang H., Xu Z., Wang H., Qaid S. M. H., Mohammed O. F., Zang Z. (2024). Iodide Management
and Oriented Crystallization Modulation for High-Performance All-Air
Processed Perovskite Solar Cells. Adv. Mater..

[66] Lin Y.-S., Abate S. Y., Wang C.-I., Wen Y.-S., Chen C.-I., Hsu C.-P., Chueh C.-C., Tao Y.-T., Sun S.-S. (2021). Low-cost
hole-transporting materials based on carbohelicene for high-performance
perovskite solar cells. ACS Appl. Mater. Interfaces.

[67] Nakka L., Cheng Y., Aberle A. G., Lin F. (2022). Analytical
review of
spiro-OMeTAD hole transport materials: paths toward stable and efficient
perovskite solar cells. Adv. Energy Sustainability
Res..

