# Supplementary material for: Enhancing Stability and Performance in Perovskite Solar Cells through Rationally Designed Phenanthro[9,10‑d]imidazole Derivatives for Tailored Interfacial Engineering
Source: ACS Appl Mater Interfaces. 2026 Jan 20;18(6):9892–904. doi: 10.1021/acsami.5c22843 (PMC12926941; doi:10.1021/acsami.5c22843)
Supplement: Supplementary file 1 [file am5c22843_si_001.pdf]

Supporting Information

Enhancing Stability and Performance in Perovskite Solar Cells through Rationally Designed Phenanthro[9,10-d]imidazole Derivatives for Tailored Interfacial Engineering

Rajarathinam Ramanujam,[a,b,c] *Zhong-En Shi,*[d] *Chien-Yu Lung,*[d] *Gebremariam Zebene Wubie,*[a] *Sie-Rong Li,*[a] *Chih-Ping Chen,*[d,e]* *Shih-Sheng Sun*[a]*

[a] Institute of Chemistry, Academia Sinica, Nankang, Taipei 11529, Taiwan, Republic of China

[b] Taiwan International Graduate Program, Sustainable Chemical Science and Technology, Academia Sinica, Nankang, Taipei 11529, Taiwan, Republic of China

[c] Department of Applied Chemistry, National Yang Ming Chiao Tung University, Hsinchu 30050 Taiwan, Republic of China

[d] Department of Materials Engineering and Organic Electronics Research Center, Ming Chi University of Technology, 84 Gunjuan Road, Taishan, New Taipei City 24301, Taiwan, Republic of China

[e] College of Engineering and Center for Sustainability and Energy Technologies, Chang Gung University, Taoyuan City 33302, Taiwan, Republic of China

Corresponding authors:

Email: *sssun@chem.sinica.edu.tw* (S.-S. Sun); *cpchen@mail.mcut.edu.tw* (C.-P. Chen)

Table of Contents

Measurements.. S6
Device Fabrication Methods.. S7
SCLC measurements: S8
Materials and Synthesis Procedures S9
Table S1. Summary of the best photovoltaic performance of the PSCs in literature with imidazole derivatives employed as dopant-free hole-transporting or interfacial layer (IFL) materials.. S10

Table S2. Summary of the best photovoltaic performance of the NiO_x-based inverted PSCs using organic small molecules (OSMs) and polymer (Poly)-interfacial layer (IFL) materials.. S11

Table S3. The energy levels of NiO_x films with and without SR-molecules..................... S13

Table S4. The full width at half maximum (FWHM) of ITO/NiO_x/SR-molecules/PVKs calculated from PXRD results in **Figure 5(b).** S13

Table S5. Excited-state lifetimes of perovskite films without and with SR-molecules......... S13

Table S6. The calculated values of hole mobility (μ_h), trap-filled-limit voltage (V_{TFL}) and trap-density (N_t) by SCLC method. S14

Table S7. Photovoltaic performance of champion devices with and without SR-molecules scanned from forward and reverse direction. S14

Table S8. The fitted parameters of Nyquist plots from EIS characterization.................... S15

Table S9. Selected examples of thermal stability conducted in NiO_x-based inverted PSCs in recent years. S15

Table S10. Summary of highly efficient $MAPbI_3$ based PSCs with interfacial engineering in NiO_x-HTM/perovskite interface. S17

Figure S1. ^{1}H NMR ($CDCl_3$) spectrum of compound **1**.. S18

Figure S2. ^{13}C NMR ($CDCl_3$) spectrum of compound **1**.. S18

Figure S3. ^{1}H NMR ($CDCl_3$) spectrum of **SR-1**.. S19

Figure S4. ^{13}C NMR ($CDCl_3$) spectrum of **SR-1**.. S19

Figure S5. HR-MALDI mass spectrum of **SR-1**.. S20

Figure S6. ^{1}H NMR (Acetone-d_6/$CDCl_3$ (3:1 v/v)) spectrum of **SR-2**........................... S20

Figure S7. ^{13}C NMR (Acetone-d_6/$CDCl_3$ (3:1 v/v)) spectrum of **SR-2**........................... S21

Figure S8. HR-MALDI mass spectrum of **SR-2**.. S21

Figure S9. (a) Cyclic voltammetry (CV) and (b) Differential pulse voltammetry (DPV) curves of SR-1 and SR-2 molecules in dichloromethane solutions. S22

Figure S10. (a) Thermogravimetric analysis (TGA). (b) Differential scanning calorimetry (DSC) thermograms of SR-1 and SR-2. .... S22

Figure S11. Ultraviolet photoelectron spectroscopy (UPS) spectra of (a) secondary electron cutoff and (b) onset energy regions of ITO/NiO_x, ITO/NiO_x/SR-1, and ITO/NiO_x/SR-2 films. S23

Figure S12. (a) Absorption spectra of SR-molecules spin-coated on ITO/NiO_x film and bare ITO/NiO_x film; (b) The Tauc plots derived from the absorption spectra. S23

Figure S13. The work function measured using Kelvin probe force microscopy (KPFM): (a)-(c) Contact potential difference (CPD) distributions of (a) ITO/NiO_x/PVK (control device), (b) ITO/NiO_x/SR-1/PVK, and (c) ITO/NiO_x/SR-2/PVK substrates. S24

Figure S14. FT-IR spectra of SR molecules: (a) The SR molecules drop-casted on ITO and ITO/NiO_x film (recorded using ATR-FT-IR method); (b) SR molecules grinded with PbI_2 (recorded using KBr pellet method). S24

Figure S15. Water contact angles of bare ITO/NiO_x film and SR-molecules on ITO/NiO_x film. S25

Figure S16. Tapping-mode AFM images of perovskite spin-coated on the ITO/NiO_x, ITO/NiO_x/SR-1, and ITO/NiO_x/SR-2 substrates. S25

Figure S17. Cross-sectional field-emission scanning electron microscopy (FE-SEM) images of complete device stacks: (a) ITO/NiO_x/PVK (control device), (b) ITO/NiO_x/SR-1/PVK, and (c) ITO/NiO_x/SR-2/PVK. The scale bar is 100 nm. S26

Figure S18. Tauc plots of perovskite films deposited on NiO_x, NiO_x/SR-1, and NiO_x/SR-2 layers, derived from the corresponding UV–vis absorption spectra presented in **Figure 5(a)**........... S26

Figure S19. The hole-only device configuration of control ITO/NiO_x/with and without SR-IFL/Perovskite/MoO_3/Ag for the SCLC measurements. Control device is without SR-IFL. S26

Figure S20. Schematic representation of the inverted PSCs fabricated with the configuration of ITO/NiO_x (without or with SR-IFLs)/perovskite/PCBM/BCP/Ag. S27

Figure S21. *J–V* curves hysteresis of SR-molecules in forward and reverse scans.............. S27

Figure S22. Conductivity of the devices with the configuration FTO/NiO_x (with and without SR-IFL)/Ag. S28

Figure S23. Statistics of the photovoltaic performance parameters with SR-1 (20 devices), SR-2 (16 devices), and control device (20 devices). S28

Figure S24. Steady-state photocurrent output PCE at the maximum power point (MPP) of PSCs based on SR-1(red curve) and SR-2 (blue curve). S29

Figure S25. (a) Nyquist plots of control and SR-passivated devices, (solid line = fitted with experimental data; symbols = experimental data). (b) Dark current-voltage (*J-V*) curve of devices with the control, SR-1, and SR-2 films. **…………………………………………………………** S29

Figure S26. Stability of the devices under continuous illumination (AM1.5G) with MPPT at 85–90% relative humidity. **………………………………………………………………………..** S30

References …………...…………………………………………………………………… S30

Measurements

^{1}H and ^{13}C NMR spectra were recorded on a Brüker Avance 400 NMR (400 MHz) nuclear magnetic resonance spectrometer (NMR). Mass spectra were acquired by the matrix-assisted laser desorption/ionization (MALDI) method. Absorption spectra in solutions were recorded by a using Varian Gary300 UV/Vis spectrophotometer. Corrected fluorescence spectra were measured by the Jobin–Yvon FL3-21 Horiba Fluorolog Fluorimeter. Absorption spectra of perovskite film were measured using the JASCO V-770 spectrometer. Thermogravimetric analysis (TGA) was recorded under a nitrogen atmosphere at a heating rate of 10 °C/min. Differential scanning calorimetry (DSC) was measured on a Perkin-Elmer Pyris 1.

The surface morphologies of the perovskite films were analyzed through field-emission scanning electron microscopy (FE-SEM) using a JEOL JSM 6701F. Crystallinity information was characterized using a Panalytical Empyrean X-ray diffractometer and Cu Kα (λ = 0.1542 nm) radiation. The Kelvin probe used to calculate the work function was obtained using a Kelvin probe (KP Technology). The surface roughness of the perovskites films was analyzed through atomic force microscopy (AFM), using a Brüker Dimension Edge microscope operated in the dynamic force mode at ambient temperature; the Si photomultiplier sensor exhibited a resonance frequency of 160 kHz and a force constant of 7.4 N m^{-1}. Fourier Transform Infrared (FT-IR) spectra of SR molecules were recorded in transmission and attenuated total reflectance (ATR) modes using a PerkinElmer FT-IR spectrometer. The spectra were acquired from 16 interferograms with a resolution of 4 cm^{-1}. Electrochemical impedance spectroscopy (EIS) was performed using an electrochemical workstation (IM6, Zahner) in the frequency range of 1 MHz to 1 Hz, with an amplitude of 10 mV and an applied bias of 1.1V, all in the dark. The EIS data were fitted into an

electronic circuit model using Z-View software. X-ray photoelectron spectroscopy (XPS) and ultraviolet photoelectron spectroscopy (UPS) were performed using a ULVAC-PHI PHI 5000 Versaprobe II spectrometer and a monochromatic Al Kα source. Work functions were calculated using an incident light energy of 21.2 eV [He(I) emission]. The samples were biased at –5 V dc to drive low-energy secondary electrons into the detector. Contact angles of the films were measured using a FIRST TEN ANGSTROMS FTA-1000B manual drop shape analyzer. PL spectra were acquired using an Edinburgh FLS1000 Photoluminescence Spectrometer, employing an excitation wavelength of 550 nm. TRPL spectra were recorded using a time-correlated single-photon counting spectrometer (WELLS-001 FX, DongWoo Optron). The pulse laser had a wavelength of 440 nm and an average power of 1 mW.

The photocurrent-voltage (*J-V*) characteristics of the devices were measured using a computer-controlled Keithley 2400 source measurement unit (SMU) and an Enlitech simulator (AAA Class Solar Simulators) under AM 1.5 illumination (1000 Wm^{-2}). The illumination intensity was calibrated using a standard Si reference cell and a KG-5 filter. EQEs were measured using an Enlitech QE-R spectral response measurement system to calibrate the current densities of the devices.

Device Fabrication Methods

The ITO-coated glass substrates (sheet resistance of 15 Ω sq^{-1}) were ultrasonically cleaned with abstergent aqueous solution, deionized water, acetone, and isopropyl alcohol for 20 min and then, dried with a nitrogen (N_2) stream before next use. Finally, the substrates were cleaned with air plasma for 10 min. A NiO_x film (≈20 nm) was prepared by spin-coating a solution containing NiO_x (nickel(II) acetylacetonate (55 mg) dissolved in 2.5 mL of anhydrous ethanol with the addition of 25 μL of 38% HCl). The NiO_x-coated substrates were then baked at 250 °C for 30 min and then at

300 °C for 5 min in air. SR-molecules were dissolved in 1 mg/mL of toluene. These precursor solutions were spin-coated on the NiO_x-coated substrates at 8000 rpm for 30 s in the glovebox, The $CH_3NH_3PbI_3$ precursor solution was obtained by dissolving 1.4 M of PbI_2 and MAI (molar ratio of 1:1) in anhydrous DMF: DMSO (4:1). The urea added into the precursor was dissolved in anhydrous DMF (314 mg/mL). Appropriate amounts of urea were added into the perovskite precursor solution with a concentration of 1 vol%. The solution was stirred at 60 °C for 2 h in an argon glovebox. The perovskite precursor solutions were spin coated on the SR-molecules coated substrates at Step-1 2000 rpm for 10 s and Step-2 4000 rpm 20 s in the glovebox. Subsequently, 400 μL of toluene was rapidly dropped on the substrates to induce a fast crystallization after ~6 s of spin coating. The perovskite precursor-coated substrate was dried on a hot plate at 100 °C for 10 min. Subsequently, the PCBM (20 mg/mL in anhydrous chlorobenzene) was then sequentially deposited via spin coating at 2000 rpm for 30 s; following the spin-coating deposition of BCP (0.5 mg/mL in IPA) at 6000 rpm for 30 s. Finally, the device was completed upon the evaporation of Ag contact electrodes (100 nm) at a vacuum level of 10^{-7} Pa through a shadow mask. The active area of this electrode was fixed at 0.1 cm^2. All device measurements were performed in the ambient environment (~40% humidity) at room temperature.

$$\mathrm{PCE} = \frac{J_{\mathrm{sc}} \cdot V_{\mathrm{oc}} \cdot \mathrm{FF}}{P_{\mathrm{in}}} \quad \text{----- eq. (S1)}$$

J_{sc} is the short-circuit current density (units of mA cm^{-2}), V_{oc} is the open-circuit voltage (units of V), FF is the fill factor (a unitless number), and P_{in} is the AM1.5 solar irradiation (100 mW cm^{-2}).

SCLC measurements: The space-charge-limited current (SCLC) measurement was conducted to determine the hole mobility and trap density (N_t) at the NiO_x/perovskite interface with and without

the SR-IFL. Hole-only devices were fabricated with device structures of ITO/NiO_x/with or without SR-molecules/perovskite/MoO_3/Ag.

$$J = \frac{9}{8}\varepsilon_r\,\varepsilon_0\,\mu_h\frac{V^2}{L^3} \text{ ----- eq. (S2)}$$

$$N_t = \frac{2\varepsilon_r\varepsilon_0 V_{TFL}}{qL^2} \text{ ------ eq. (S3)}$$ [S1]

The hole mobility was calculated by equation S2 where ε_r is the dielectric constant of the material, ε_0 is the permittivity of free space (8.85×10^{-12} F/m), μ_h is the hole mobility, V is the device's applied voltage, and L is the thickness of the perovskite films; the non-passivated perovskite film shows 550 nm; SR-1 and SR-2 passivated perovskite films show 580 and 560 nm of thickness, respectively. As shown in equation S3, the trap density was calculated from the so-called trap-filled-limit voltage where V_{TFL} is the trap-filled-limit voltage, q is the elementary charge, and N_t is the trap density.

Materials and Synthesis Procedures

All reagents and starting materials were commercially available without further purification, unless noted. All solvents were purified and dried by standard methods.[S2] Compounds 1 and *p*-OMeTPA were prepared according to the literature with minor modifications.[S3, S4]

Synthetic route for 5,10-dibromo-2-(4-bromophenyl)-1-phenyl-1H-phenanthro[9,10-d] imidazole **(1):**

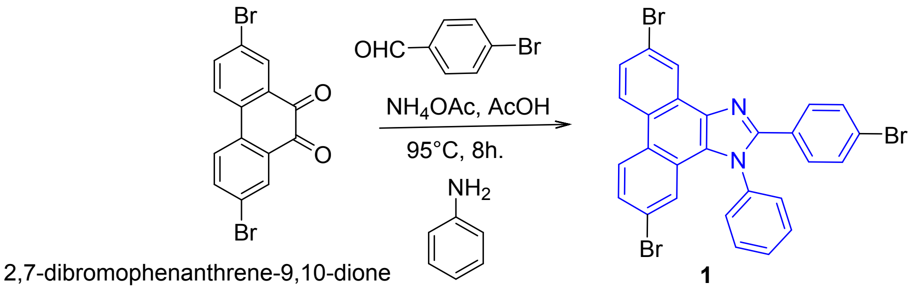

A mixture of compound 2,7-dibromophenanthrene-9,10-dione (2 g, 5.46 mmol), 4-bromobenzaldehyde (1.0 g, 5.46 mmol), 4-bromoaniline (1.4 g, 8.19 mmol), and ammonium acetate (1.05 g, 13.65 mmol) in 40 mL of acetic acid was heated at 95 °C for 8 h. After being cooled to room temperature, the reaction mixture was quenched in cold water. After stirring for 10 minutes, the product precipitated, which was filtered and washed with ethanol to give a white powder of compound **2** with a yield of 84%. ^1H NMR (400 MHz, $CDCl_3$, δ): 8.97 (d, J = 2.16 Hz, 1H), 8.50 (d, J = 8.92 Hz, 1H), 8.45 (d, J = 8.90 Hz, 1H), 7.74-7.65 (m, 3H), 7 .66 (s, 1H), 7.57 (dd, J = 8.92, 1.99 Hz, 1H), 7.49 (dd, J = 7.79, 1.08 Hz, 2H), 7.45-7.42 (m, 4H), 7.18 (d, J = 1.97 Hz, 1H). ^{13}C NMR (100 MHz, $CDCl_3$): δ 150.4, 137.8, 137.2, 131.6, 130.7, 130.6, 130.4, 129.1, 128.9, 128.8, 128.4, 128.3, 127.7, 127.4, 126.5, 125.6, 125.4, 124.8, 124.1, 123.8, 123.7, 122.2, 121.0.

Table S1. Summary of the best photovoltaic performance of the PSCs in literature with imidazole derivatives employed as dopant-free hole-transporting or interfacial layer (IFL) materials

Device Configuration	Function of imidazole derivatives (dopant-free condition)	Control Device PCE (η %) /Champion device (η %)	Reference/year
ITO/NiO_x/ME2/$CH_3NH_3PbI_3$/PCBM/BCP/Ag	IFL	16.3/19.22	Ref[S5]/2018
ITO/NiO_x/TPI6MEO/$CH_3NH_3PbI_3$/PCBM/BCP /Ag	IFL	14.57/18.42	Ref[S6]/2019
ITO/AI110/$CH_3NH_3PbI_3$/PCBM/BCP/Ag	HTM[a]	12.55/14.9	Ref[S7]/2021
FTO/TiO_2/$CH_3NH_3PbI_3$/Ph-TPA-4A/MoO_3/Ag	HTM	16.8[b]/18.03	Ref[S8]/2019

ITO/ TiO_2/SnO_2/PCBM/$FA_{0.84}MA_{0.16}Pb(I_{1-x}Br_x)_3$/3-ImBT-2D/Au	HTM	22.53[b]/17.79	Ref[S9]/2023
ITO/AI112/$CH_3NH_3PbI_3$/PCBM/BCP/Ag	HTM	11.54/11.98	Ref[S10]/2020
ITO/PI2/$FA_{0.85}MA_{0.15}PbI_3$/PCBM/BCP/Ag	HTM	19.01/19.11	Ref[S11]/2021
ITO/2-TPA-PI/$CH_3NH_3PbI_3$/PCBM/BCP/Ag	HTM	14.11/17.58	Ref[S12]/2023
ITO/NiO_x/organic-HTM/ $Cs_{0.04}(FA_{0.92}MA_{0.08})_{0.96}Pb(I_{0.92}Br_{0.08})_3$/PCBM /BCP/Ag.	IFL	16.07/17.22	Ref[S13]/2023
FTO/DIM/$FA_{0.75}MA_{0.15}Cs_{0.05}PbI_{2.7}Br_{0.3}$/ $PC_{61}BM$/BCP/Ag.	HTM	13.63/19.15	Ref[S14]/2024
ITO/NiO_x/SR-1/$CH_3NH_3PbI_3$/PCBM/BCP/Ag	IFL	17.4/20.3	This Work

[a]HTM = Hole transporting material. [b] The control device is based on with dopant condition.

Table S2. Summary of the best photovoltaic performance of the NiO_x-based inverted PSCs using organic small molecules (OSMs) and polymer (Poly)-interfacial layer (IFL) materials

Device Configuration	OSMs or polymer	Control Device PCE (η %) /Champion device (η %)	Reference/year
ITO/NiO_x/6FPPY/PVK/PCBM/BCP/Ag	OSM-IFL	22.2/24.5	Ref[S15]/2023
ITO/NiOx/QxNN/$Cs_{0.16}FA_{0.80}MA_{0.04}Pb(I_{0.68}Br_{0.32})_3$/$C_{60}$/BCP/Ag	OSM-IFL	17.5/20.0	Ref[S16]/2024
ITO/NiOx/2DPP/$CH_3NH_3PbI_3$/$PC_{61}BM$/BCP/Ag	OSM-IFL	19.38/21.9	Ref[S17]/2024
ITO/NiO_x/BTF14/$FAPbI_3$/PCBM/BCP/Ag	OSM-IFL	22.11/24.2	Ref[S18]/2024
ITO/NiOx/WH14/ $Cs_{0.05}FA_{0.995}PbI_3$/C_{60}/BCP/Ag	OSM-IFL	23.7/25.2	Ref[S19]/2025
ITO/NiOx/SPPaTFA/$Cs_{0.05}(FA_{0.95}MA_{0.05})_{0.95}Pb(I_{0.95}Br_{0.05})_3$/$PC_{61}BM$/BCP/Ag	OSM-IFL	23.38/25.54	Ref[S20]/2025

ITO/NiO_x/Cor-AI/ $Cs_{0.05}(FA_{0.95}MA_{0.05})_{0.95}Pb(I_{0.95}Br_{0.05})_3$PCBM/BCP/ Ag	OSM-IFL	19.17/23.01	ref [S21]/2025
FTO/NiO_x/MeO-4PADBC/$Cs_{0.05}FA_{0.85}MA_{0.1}PbI_3$/$CF_3$-PEAI/PCBM/BCP/Ag	[a]SAM-IFL	24.1/25.6	Ref[S22]/2023
ITO/NiO_x/TBT-BA/ $Cs_{0.04}(FA_{0.96}MA_{0.04})_{0.96}Pb(I_{0.96}Br_{0.04})_3$ /PEAI/PCBM/BCP/Ag	SAM-IFL	20.8/24.8	Ref[S23]/2024
ITO/NiO_x/2PACz/D3-PACz w/ and w/o /$FAPbI_3$/PCBM/BCP/Ag	SAM-IFL	21.7/23.8	Ref[S24]/2024
FTO/ALD-NiO_x/3PATAT/$FAPbI_3)_{0.9}(CsPbI_3)_{0.05}$-$(MAPbBr_3)_{0.05}$/$C_{60}$/BCP/Ag	SAM-IFL	21.1/25.1	Ref[S25]/2025
ITO/NiO_x/Me-4PACz/4Br-BPA(w/ and w/o)/ $FA_{0.85}MA_{0.1}Cs_{0.05}PbI_3$/$C_{60}$/BCP/Ag	[b]Co-SAM-IFL	25.05/26.59	Ref[S26]/2024
ITO/NiO_x/S-BA-SAM:Me-4PACz/$FA_{0.75}MA_{0.15}Cs_{0.05}PbI_{2.7}Br_{0.3}$/$PC_{61}BM$/$SnO_2$/Ag.	Co-SAM-IFL	18.9/20.1	Ref[S27]/2025
ITO/NiO_x/Me-4PACz/CzTPA w/ and w/o /$FA_{0.95}Cs_{0.05}PbI_3$/C_{60}/SnO_2/Ag	Co-SAM-IFL	23.53/25.66	Ref[S28]/2025
FTO/NiO_x/MeO-2PACz/ Cl-OCZ or Br-OCZ w/ and w/o / RbCsFAMA-perovskite/PCBM/BCP/Ag	Co-SAM-IFL	25.26/26.57	Ref[S29]/2025
FTO/NiO_x/PTAA/Al_2O_3/K_δ-CsFAMA-perovskite/PEAI/PCBM/BCP/ALD-SnO_2/Ag	Poly-IFL	18.23/21.9	Ref[S30]/2021
FTO/NiO_x/PMD25/$CH_3NH_3PbI_3$/$PC_{61}BM$/BCP/ Ag	Poly-IFL	15.64/19.56	Ref[S31]/2023
ITO/NiO_x/PB2T-E/A/$Cs_{0.05}FA_{0.95}PbI_3)_{0.97}(MAPbBr_3)_{0.03}$/FMG/BCP/Ag	Poly-IFL	20.65/23.22	Ref[S32]/2024
PET/ITO/NiO_x/Poly(FB-TA)/$Cs_{0.05}(FA_{0.92}MA_{0.08})_{0.95}Pb(I_{0.97}Br_{0.03})_3$/ Poly(FB-TA)/PCBM /BCP/Ag.	Poly-IFL	19.38/21.42	Ref[S33]/2024

[a]SAM = Self-Assembled Monolayers; [b]Co-SAM = Co-adsorbed SAM.

Table S3. The energy levels of NiO_x films with and without SR-molecules

films	Valence band maximum, E_{VB} (eV)	Energy gap, E_g (eV)	Conduction band minimum, E_{CB} (eV)
ITO/NiO_x	-5.27	-3.86	-1.41
ITO/NiO_x/SR-1	-5.19	-3.83	-1.36
ITO/NiO_x/SR-2	-5.24	-3.82	-1.42
$MAPbI_3$	-5.40	-1.60	-3.80

Table S4. The full width at half maximum (FWHM) of ITO/NiO_x/SR-molecules/PVKs calculated from PXRD results in **Figure 5(b).**

Planes		Control device	SR-1	SR-2
(110)	FWHM	0.4223	0.3706	0.3702
(220)	FWHM	0.4014	0.3857	0.3894
(310)	FWHM	0.3923	0.3820	0.3948

Table S5. Excited-state lifetimes of perovskite films without and with SR-molecules

Films	τ1 [ns]	τ2 [ns]	$\tau_{average}$ [ns]
Control	14.1	65.3	39.7
With SR-1	3.1	12.6	7.9
With SR-2	3.3	13.7	8.5

Table S6. The calculated values of hole mobility (μ_h), trap-filled-limit voltage (V_{TFL}) and trap-density (N_t) by SCLC method.

	V_{TFL} (V)	L (m)	ε_r (F/m)	ε_0 (F/m)	q (C)	N_t (cm^{-3})	μ_h ($cm^2V^{-1}s^{-1}$)
Control	0.41	5.5×10^{-7}				3.81×10^{15}	1.27×10^{-3}
With SR-1	0.23	5.8×10^{-7}	25	8.85×10^{-12}	1.69×10^{-19}	1.86×10^{15}	1.61×10^{-3}
With SR-2	0.25	5.6×10^{-7}				2.18×10^{15}	1.49×10^{-3}

L = thickness of passivated or non-passivated perovskite film; ε_r = dielectric constant; ε_0 = permittivity of free space; q = elementary charge.

Table S7. Photovoltaic performance of champion devices with and without SR-molecules scanned from forward and reverse direction.

Substrates	V_{oc} (V)	J_{sc} (mA cm^{-2})	FF (%)	PCE (%)	[a]HI (%)
SR-1-FS	1.07	23.4	80.0	20.0	
SR-1-RS	1.09	23.5	79.2	20.3	1.3
SR-2-FS	1.07	22.5	79.4	19.1	
SR-2-RS	1.06	23.2	80.8	19.9	3.8
Control-FS	1.05	21.2	73.3	16.3	
Control-RS	1.09	21.4	74.4	17.4	6.3

[a]$HI = \frac{PCE_{RS}-PCE_{FS}}{PCE_{RS}} \times 100$, where HI = hysteresis index.

Table S8. The fitted parameters of Nyquist plots from EIS characterization[a]

Substrates	R_s (Ω)	R_{rec} (Ω)	C_{rec} (nF)
SR-1	1.96	743	64.4
SR-2	3.67	724	79.8
Control	4.88	428	110

[a] R_s = series of resistance, and R_{rec} = charge recombination resistance.

Table S9. Selected examples of thermal stability conducted in NiO_x-based inverted PSCs in recent years.

**Device Configuration (p-i-n)**	**Thermal Stability**	**MPP tracking**	**Reference / year**
ITO/NiO_x/$Cs_{0.05}MA_{0.1}FA_{0.85}PbI_3$/3F-PEAI/PCBM/$SnO_2$/Au	Unencapsulated device PCE retained 92% for 500 h/65 °C under N_2 atm with RH of 50%.	MPPT	2022[34]
FTO/NiO_x/$(MA_{0.17}FA_{0.83})_{0.95}Cs_{0.05}Pb(I_{0.9}Br_{0.1})_3$/PCBM/BCP/Cr/$Cr_2O_3$/Au	Encapsulated device PCE retained 80% for 1072 h/70-75 °C under sunlight with RH of 40-50%.	NO	2019[35]
ITO/NiO_x/PTAA/$CH_3NH_3PbI_3$/PCBM/BCP/Ag	Unencapsulated device PCE retained 90% for 200 h/65 °C under N_2 atm.	NO	2022[36]
	Unencapsulated device PCE retained 87% for 200 h under ambient air with RH of 43%..	MPP	
FTO/ALD-NiO_x/$Cs_{0.05}MA_{0.95}PbI_3$/PCBM/BCP/ALD-AZO/Al	PCE retained 86.7% for 500 h/85 °C, under ambient air with RH of 20-60%.	MPPT	2018[37]
	Unencapsulated device PCE retained 98% for 350 h/65 °C under N_2 atm.	NO	2024[38]

ITO/NiO_x/MeO-2PACz(SAM)/FACsPb(I_xBr_x)$_x$/PCBM/BCP/Ag	Unencapsulated device, PCE retained 99% for 340 h/25 °C under N_2 atm.	MPPT	
FTO/NiO_x/AlO_x/$Cs_{0.05}MA_{0.95}PbI_3$/PCBM/BCP/ALD-AZO/Al	Encapsulated device PCE retained 90% for 2000 h/85 °C under ambient air with RH of 50%.	MPPT	2024[S39]
ITO/NiO_x/Triton/$Cs_{0.05}(MA_{0.15}FA_{0.85})_{0.95}Pb(I_{0.85}Br_{0.15})_3$/PCBM/Ag	PCE retained 69.0% for 760 h/85 °C under N_2 atm with RH of 40-50%	NO	2023[S40]
ITO/NiO_x/TPA-BA (SAM)/CsFAMA/PCBM/BCP/Ag	Unencapsulated device PCE retained 90% for 1000 h/85 °C under N_2 atm.	NO	2022[S41]
ITO/NiO_x/TBT-BA (SAM)/$Cs_{0.04}(FA_{0.96}MA_{0.04})_{0.96}Pb(I_{0.96}Br_{0.04})_3$/PEAI/PCBM/BCP/Ag	Unencapsulated device PCE retained 88.7% for 2635 h/60 °C under N_2 atm.	NO	2024[S23]
	Unencapsulated device PCE retained 85.8% for 200 h/85 °C under N_2 atm.	NO	
	Encapsulated device PCE retained 81.2% for 240 h/30 °C under N_2 atm with RH of 45-55%	MPPT	
ITO/NiO_x/PTAA/$FA_{0.95}Cs_{0.05}PbI_3$/PCBM:DBCP/BCP/Ag	Under one-sun illumination of unencapsulated device, PCE retained 95% for 2500 h.	MPPT	2024[S42]
	Thermal stability of Encapsulated, PCE retained 90% for 1500 h/85 °C with RH of 85%.	No	
ITO/NiO_x/MeO-2PACz(SAM)/Al_2O_3/ **α**-$FAPbI_3$:BLS(*doping*)/PCBM/BCP/Ag	Thermal stability of unencapsulated device PCE retained 80% for 500 h/80 °C with RH of 40-50%.	NO	2024[S43]
	Under one-sun illumination of unencapsulated device, PCE retained 96% for 1243h under N2 atm.	MPPT	
ITO/NiO_x/SR-1/$MAPbI_3$/PCBM/BCP/Ag	Unencapsulated device PCE retained 97.4% for 350 h/60 °C under N_2 atm with RH of 50-60%.	NO	**This Work**
	Under one-sun illumination of unencapsulated device, PCE retained 96% for 2000 s with RH of 85-90%.	MPPT	

Table S10. Summary of highly efficient $MAPbI_3$ based PSCs with interfacial engineering in NiO_x-HTM/perovskite interface.

Device Configuration (p-i-n)	NiOx/interlayer or dopant/PVKs	Control Device PCE (η %) /Champion device (η %)	Stability/Test condition	Reference/year
ITO/NiO_x/$CH_3NH_3PbI_3$/PCBM/BCP/Ag	NiO_x/PTAA	15.19/ 19.92	Unencapsulated device PCE retained 90% for 8 d/65 °C N_2 atm	Ref[S36]/2022.6
ITO/NiO_x/$CH_3NH_3PbI_3$/PCBM/BCP/Ag	NiO-stabilizer	13.29/ 19.91	Encapsulated device PCE retained 97% for 33 d/under air	Ref[S44]/2022.7
ITO/NiO_x/$CH_3NH_3PbI_3$/$PC_{61}BM$/BCP/Ag	NiOx/CBSA (SAMs)	18.2/ 20.7	Encapsulated device PCE retained 84% for 125 d/ 25 °C N_2 atm and 80% for 42 d under air /RH of 50–70%, 25 °C	Ref[S45]/2022.5
ITO/NiO_x/$CH_3NH_3PbI_3$/$PC_{61}BM$/BCP/Ag	NiO_x-metal dopant (Cu)	18.18/ 20.26	Encapsulated device PCE retained 95% for 42 d/RH of 50–65%, 23 °C, under N_2 atm	Ref[S46]/2018.7
FTO/NiO_x/$CH_3NH_3PbI_3$/$PC_{61}BM$/BCP/Ag	NiO_x-boric acid dopant	18.71/21.4	Unencapsulated device PCE retained 92.8% for 58 d/ RH of 10–20%, 20-30 °C, Under light illumination ($10 mWcm^{-2}$)	Ref[S47]/2021.4
ITO/NiO_x/$CH_3NH_3PbI_3$/$PC_{61}BM$/ AM-TiO_x/Ag	NiO_x-metal dopant (Al)	16.56/20.84	Unencapsulated device PCE retained 84.3% for 72 d/ (RH 55-60%, Under N_2 atm)	Ref[S48]/2020.5
ITO/NiO_x/$CH_3NH_3PbI_3$/PCBM/BCP/Ag	NiO_x/TRUX-D1 (OSMs)	17.3/ 20.8	Unencapsulated device PCE retained 75.5% for 80 d under air/RH 40%, 25 °C; 98% for 210 d/under N_2 atm	Ref[S49]/2024.3
ITO/NiO_x/$CH_3NH_3PbI_3$/PCBM/BCP/Ag	NiO_x/SR-1 (OSMs)	17.4/ 20.3	Unencapsulated device PCE retained 97.4% for 350 h/60 °C under N_2 atm with RH of 50-60%.	**This work**

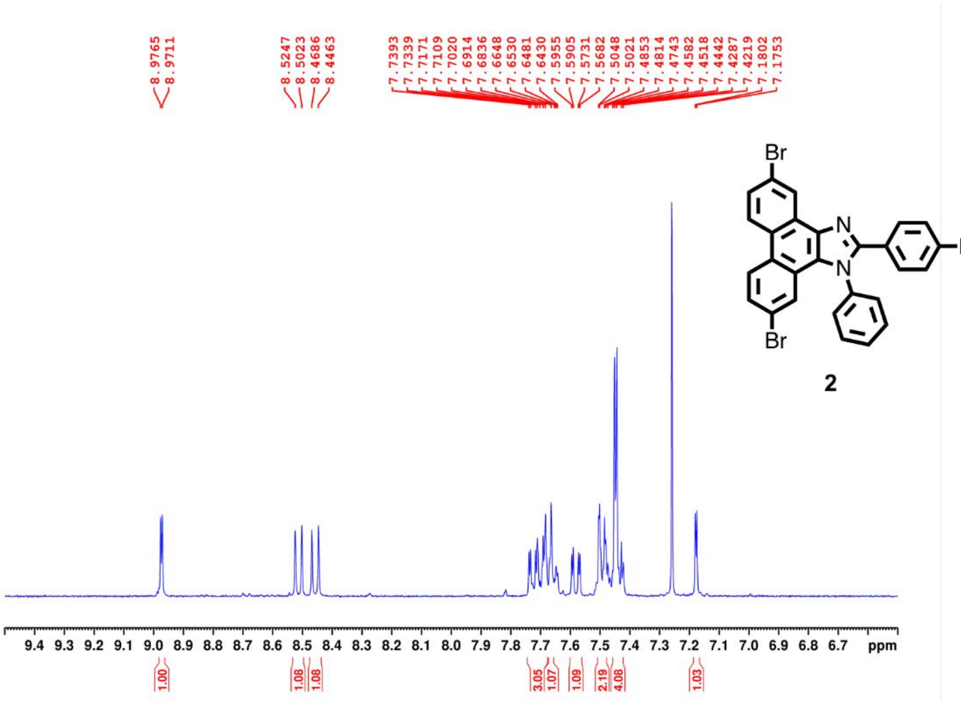

Figure S1. ^{1}H NMR ($CDCl_3$) spectrum of compound **1**

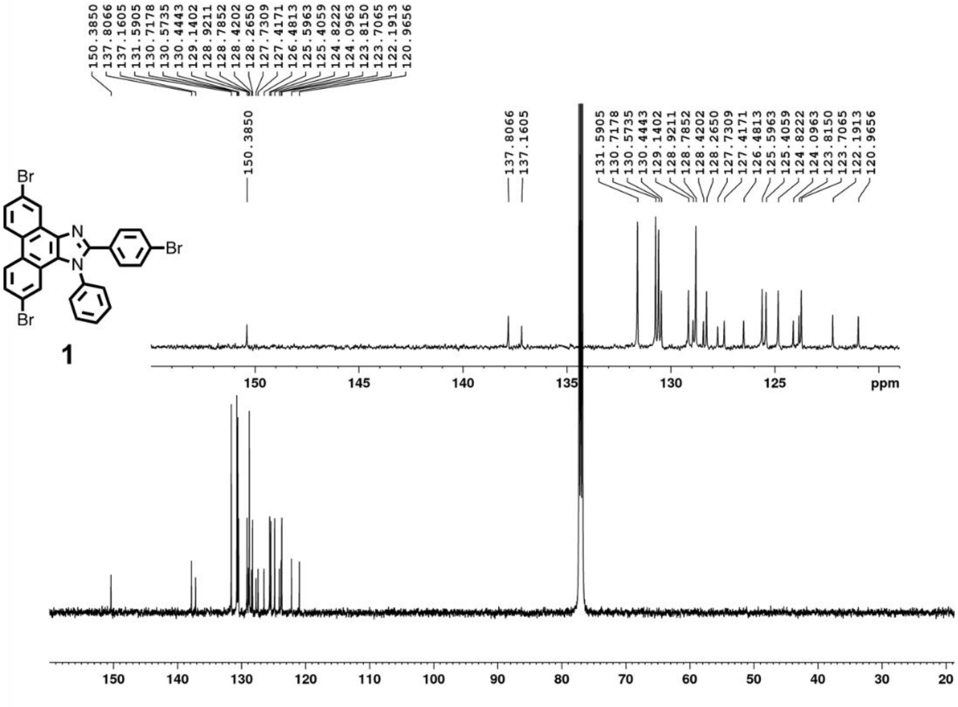

Figure S2. ^{13}C NMR ($CDCl_3$) spectrum of compound **1**

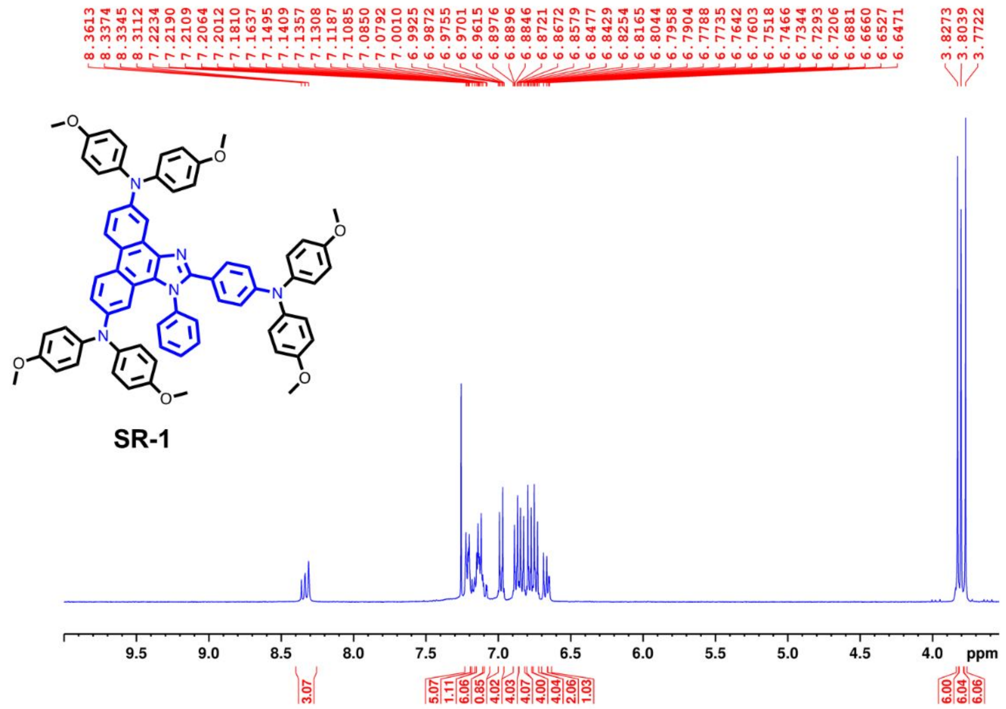

Figure S3. ^{1}H NMR ($CDCl_3$) spectrum of **SR-1**

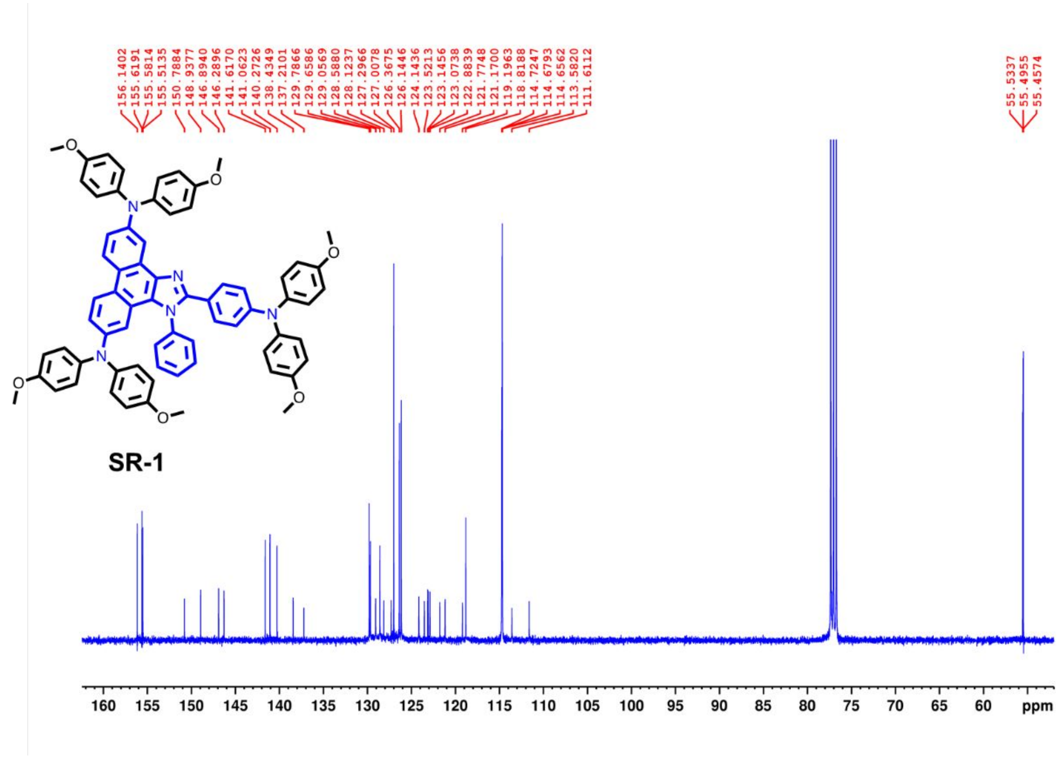

Figure S4. ^{13}C NMR ($CDCl_3$) spectrum of **SR-1**

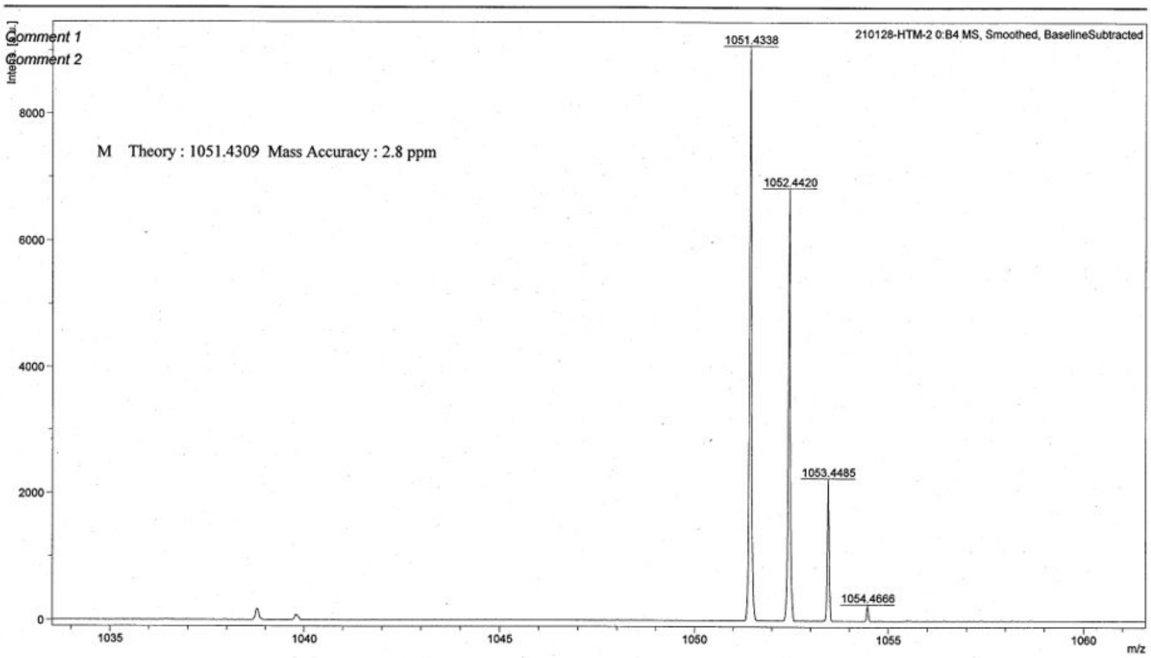

Figure S5. HR-MALDI mass spectrum of **SR-1**

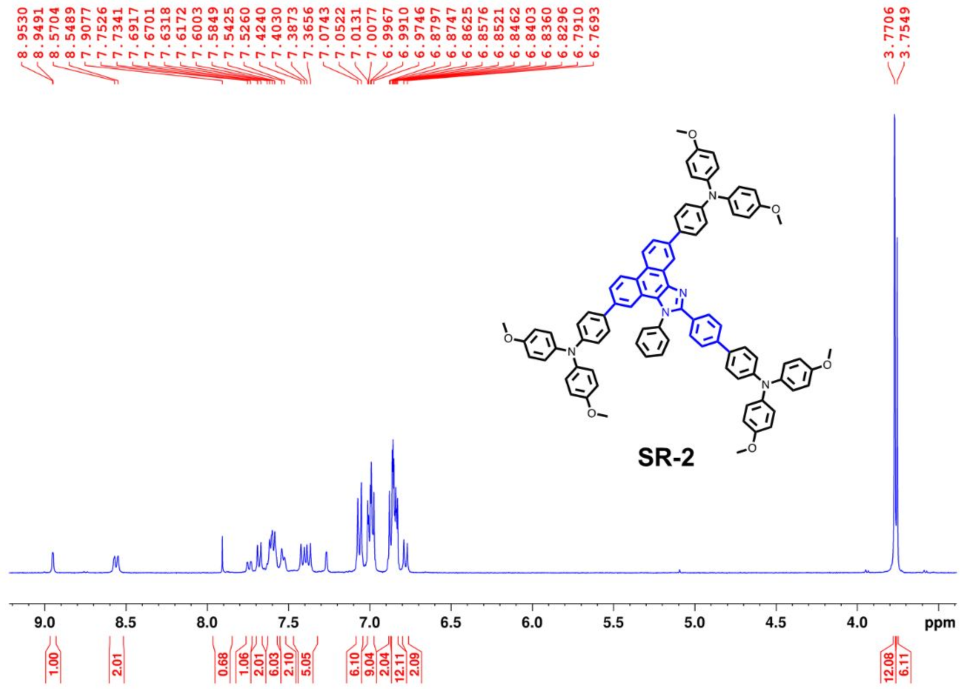

Figure S6. ¹H NMR (Acetone-d_6/$CDCl_3$ (3:1 v/v)) spectrum of **SR-2**

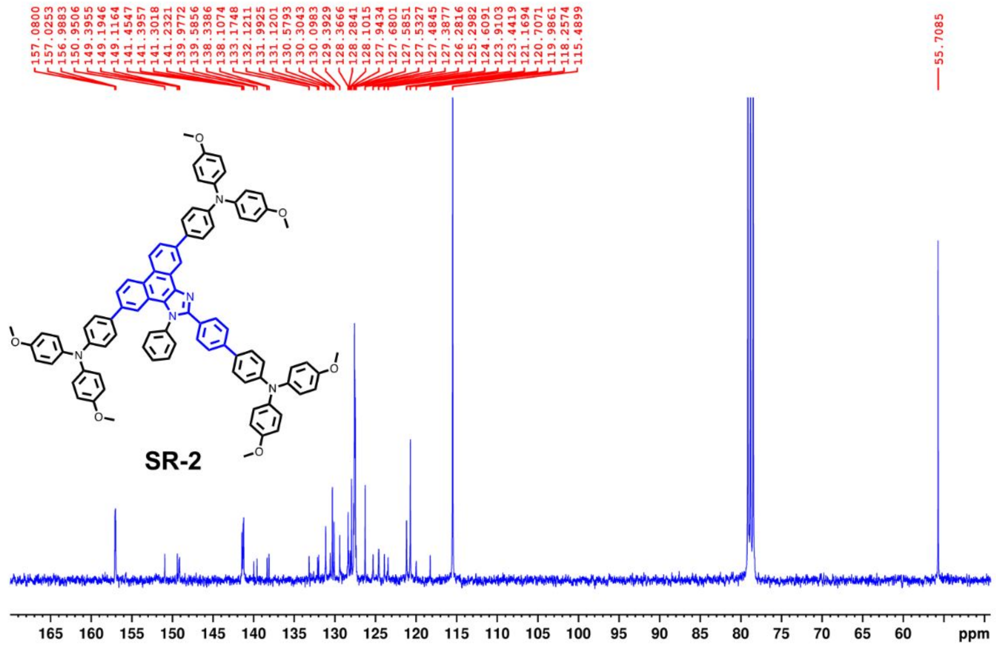

Figure S7. ^{13}C NMR (Acetone-d_6/$CDCl_3$ (3:1 v/v)) spectrum of **SR-2**

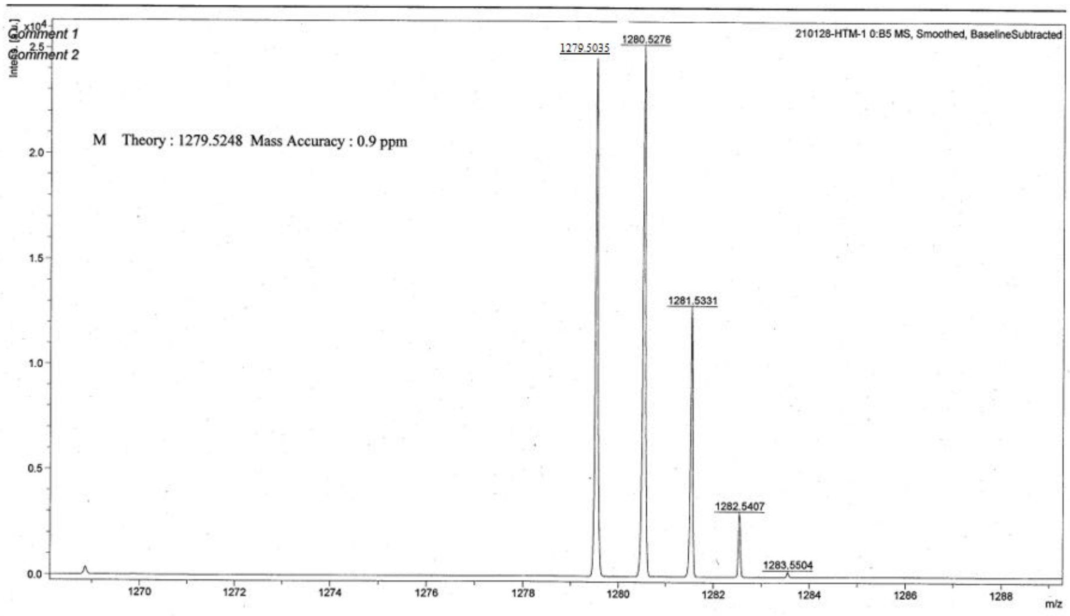

Figure S8. HR-MALDI mass spectrum of **SR-2**

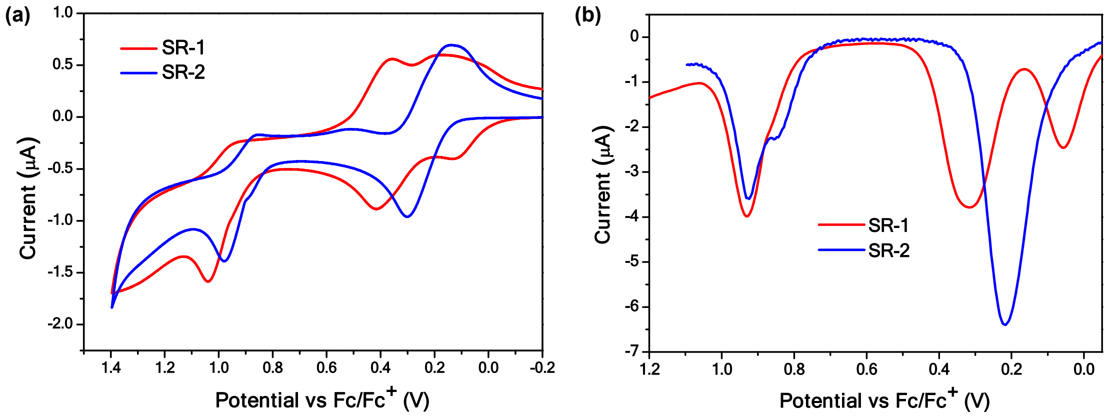

Figure S9. (a) Cyclic voltammetry (CV) and (b) Differential pulse voltammetry (DPV) curves of SR-1 and SR-2 molecules in dichloromethane solutions.

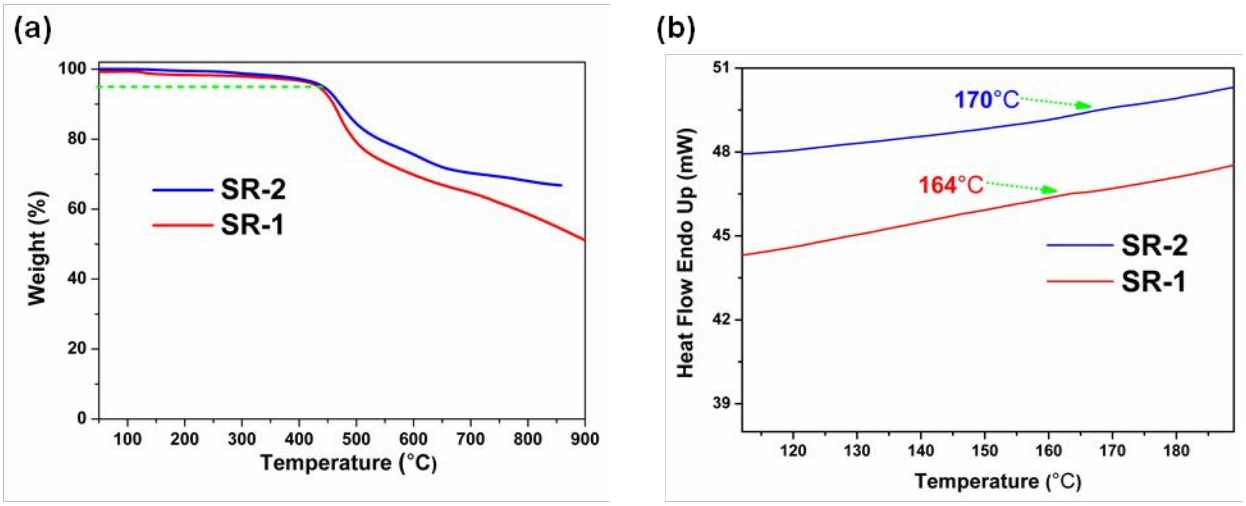

Figure S10. (a) Thermogravimetric analysis (TGA). (b) Differential scanning calorimetry (DSC) thermograms of SR-1 and SR-2.

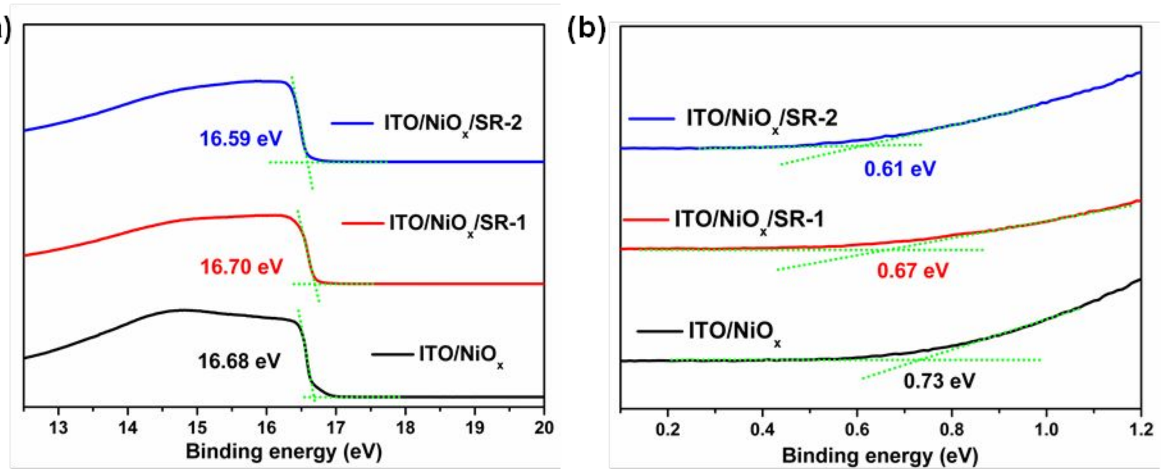

Figure S11. Ultraviolet photoelectron spectroscopy (UPS) spectra of (a) secondary electron cutoff and (b) onset energy regions of ITO/NiO_x, ITO/NiO_x/SR-1, and ITO/NiO_x/SR-2 films.

$$E_{VB} = 21.2 - (E_{cutoff} - E_{onset}) \text{ ------------------ eq. (S4)}$$

ITO/NiO_x → E_{VB} = 21.22-(16.68-0.73) = 21.22- 15.95 = 5.27 eV

ITO/NiO_x/SR-1 → E_{VB} = 21.22-(16.70-0.67) = 21.22- 16.03 = 5.19 eV

ITO/NiO_x/SR-2 → E_{VB} = 21.22-(16.59-0.61) = 21.22- 15.98 = 5.24 eV

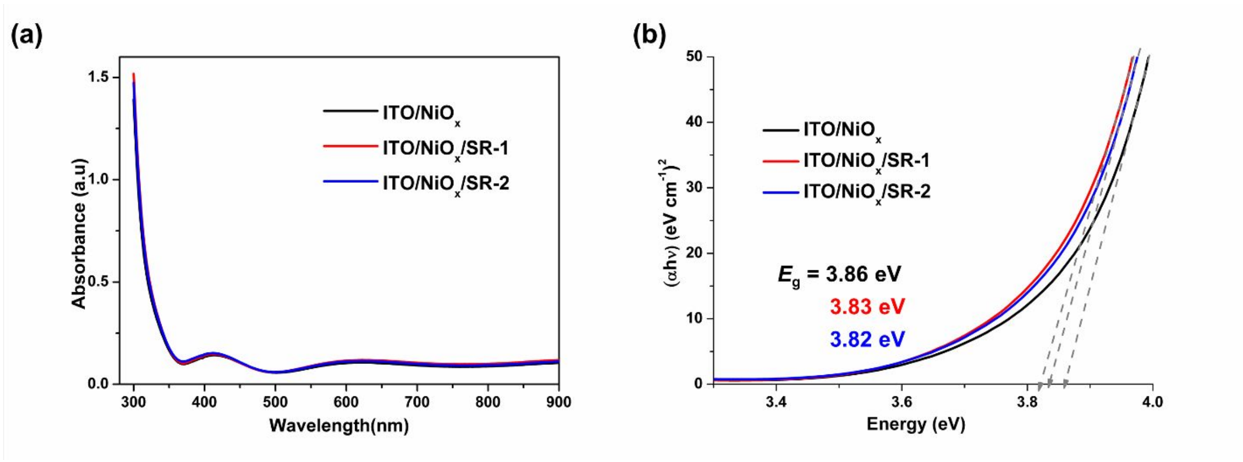

Figure S12. (a) Absorption spectra of SR-molecules spin-coated on ITO/NiO_x film and bare ITO/NiO_x film; (b) The Tauc plots derived from the absorption spectra.

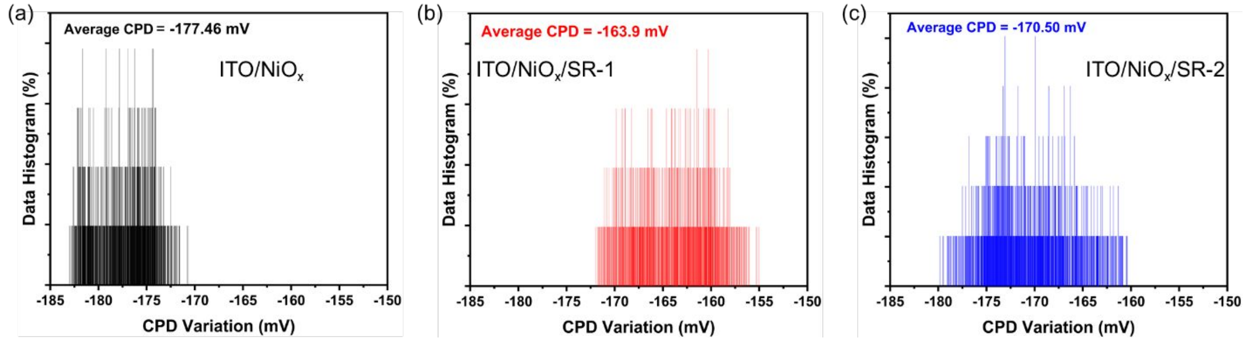

Figure S13. The work function measured using Kelvin probe force microscopy (KPFM): (a)-(c) Contact potential difference (CPD) distributions of (a) ITO/NiO_x/PVK (control device), (b) ITO/NiO_x/SR-1/PVK, and (c) ITO/NiO_x/SR-2/PVK substrates.

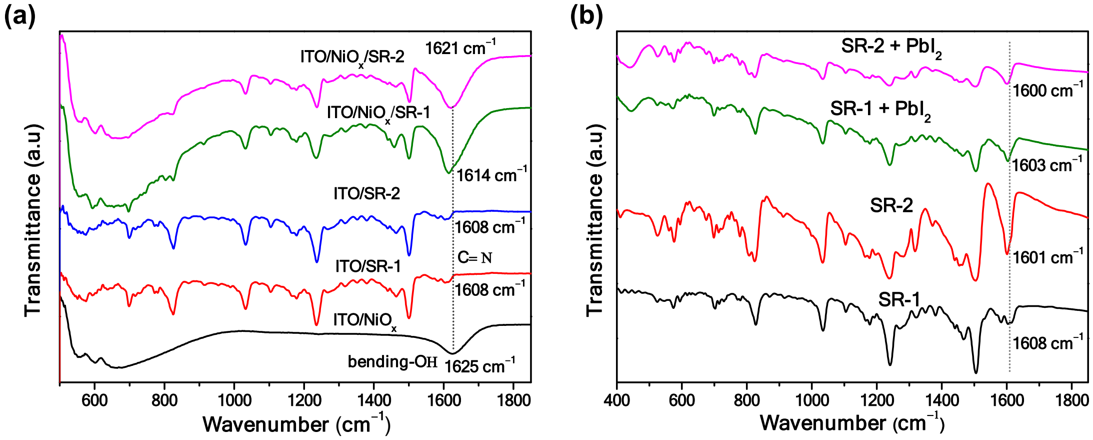

Figure S14. FT-IR spectra of SR molecules: (a) The SR molecules drop-casted on ITO and ITO/NiO_x film (recorded using ATR-FT-IR method); (b) SR molecules grinded with PbI_2 (recorded using KBr pellet method).

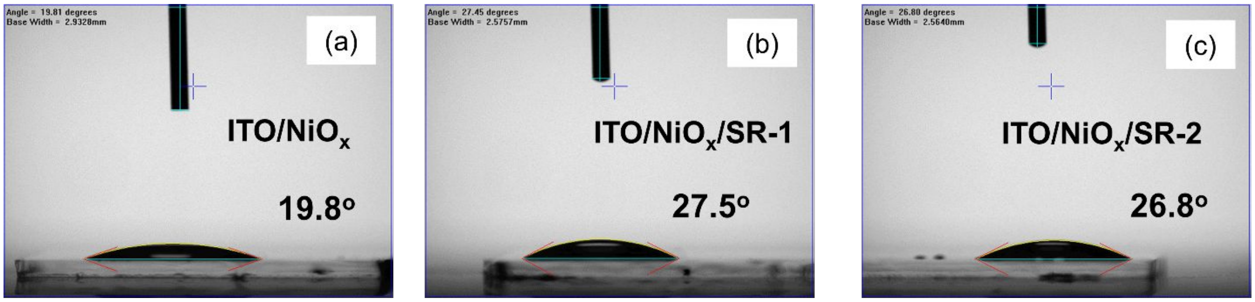

Figure S15. Water contact angles test of ITO/NiO$_x$ film with and without SR-molecules: (a) ITO/NiO$_x$, (b) ITO/NiO$_x$/SR-1, and (c) ITO/NiO$_x$/SR-2 film.

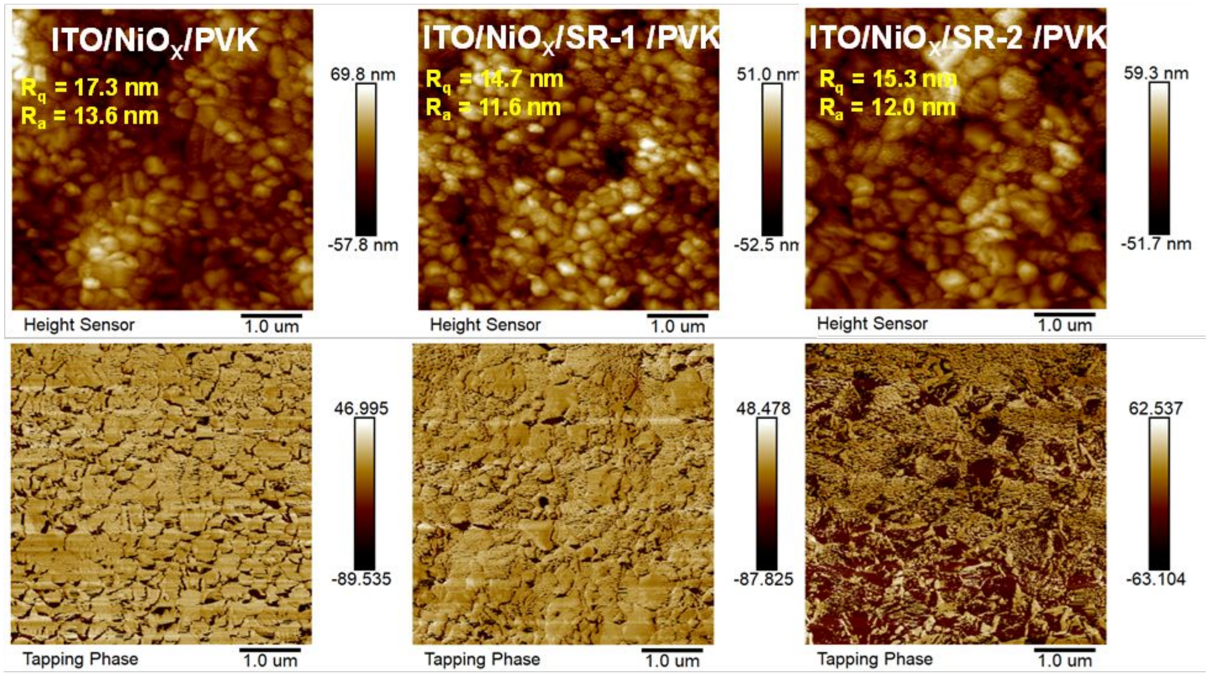

Figure S16. Tapping-mode AFM images of perovskite spin-coated on the ITO/NiO$_x$, ITO/NiO$_x$/SR-1, and ITO/NiO$_x$/SR-2 substrates.

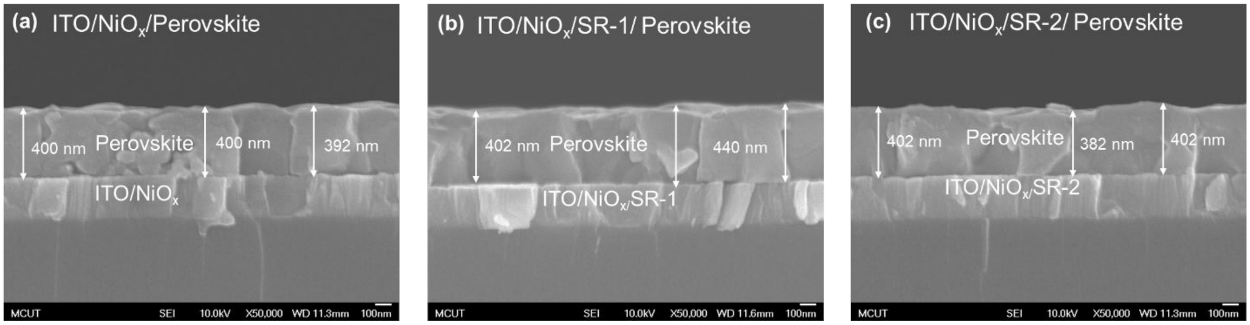

Figure S17. Cross-sectional field-emission scanning electron microscopy (FE-SEM) images of complete device stacks: (a) ITO/NiO_x/PVK (control device), (b) ITO/NiO_x/SR-1/PVK, and (c) ITO/NiO_x/SR-2/PVK. The scale bar is 100 nm.

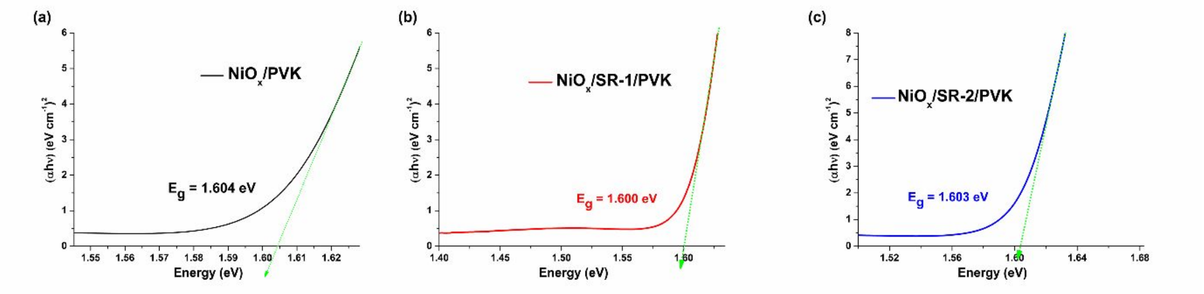

Figure S18. Tauc plots of perovskite films deposited on NiO_x layer with and without SR-molecules: (a) NiO_x/PVK, (b) NiO_x/SR-1/PVK, and (c) NiO_x/SR-2/PVK layers, derived from the corresponding UV–vis absorption spectra presented in **Figure 5(a)**.

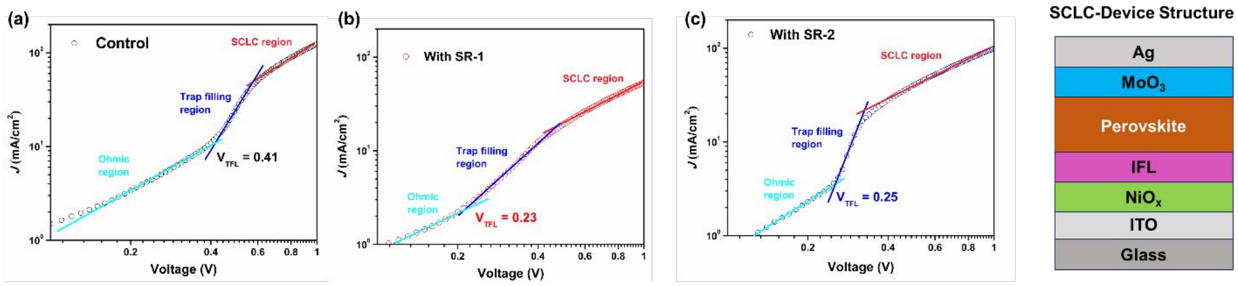

Figure S19. The hole-only device configuration: ITO/NiO_x/with and without SR-IFL/Perovskite/MoO_3/Ag for the SCLC measurements. (a) Control, (b) with SR-1, and (c) with SR-2.

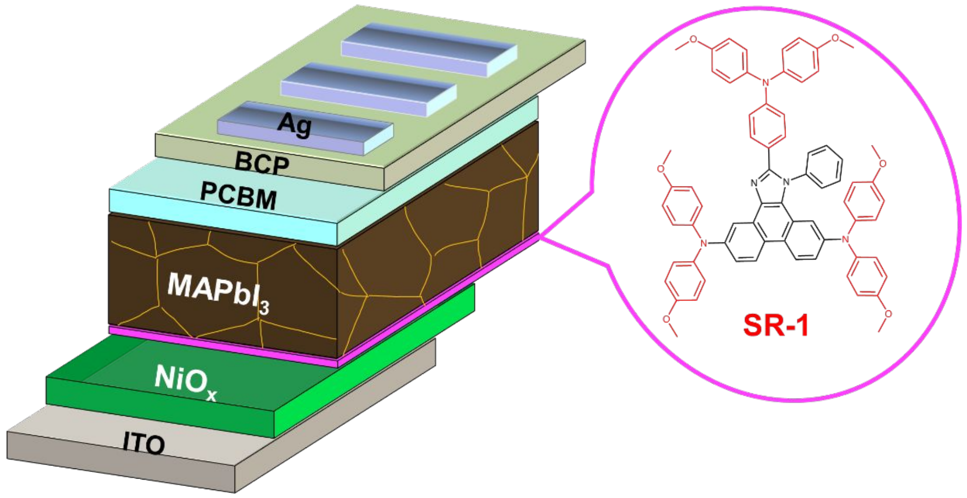

Figure S20. Schematic representation of the inverted PSCs fabricated with the configuration of ITO/NiO_x (without or with SR-IFLs)/perovskite/PCBM/BCP/Ag.

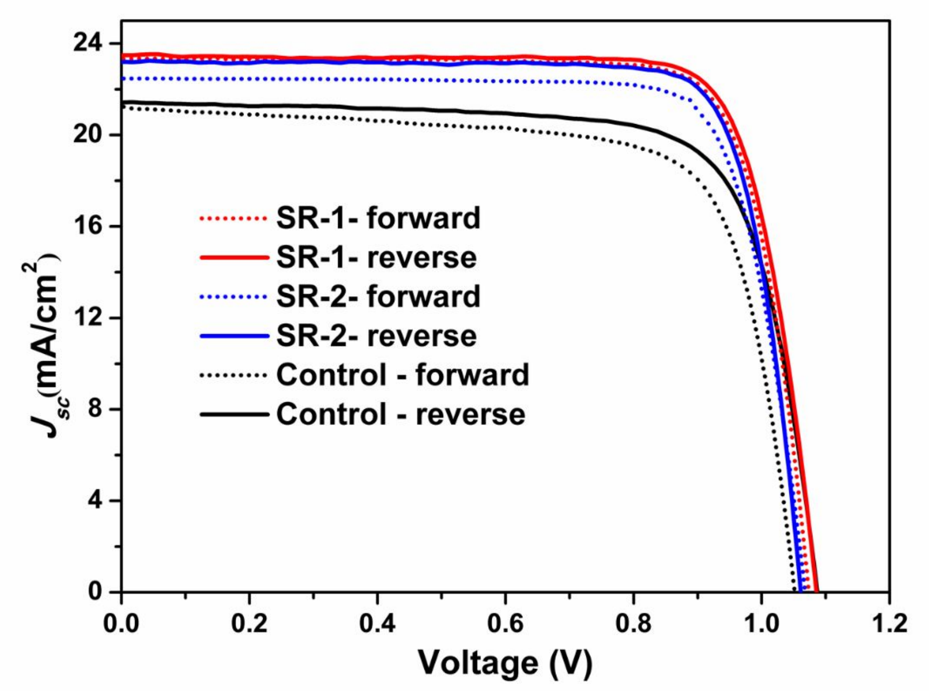

Figure S21. *J*–*V* curves hysteresis of SR-molecules in forward and reverse scans

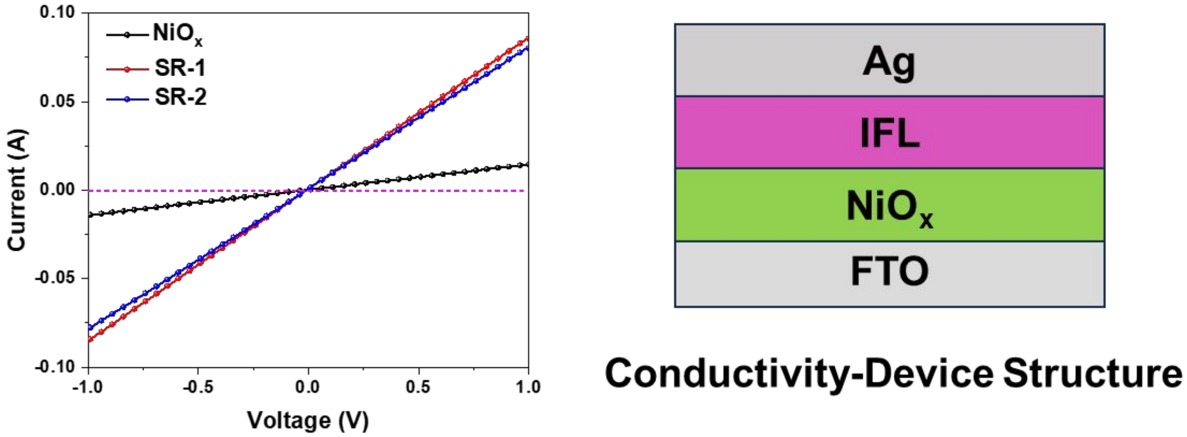

Figure S22. Conductivity of the devices with the configuration FTO/NiO_x (with and without SR-IFL)/Ag.

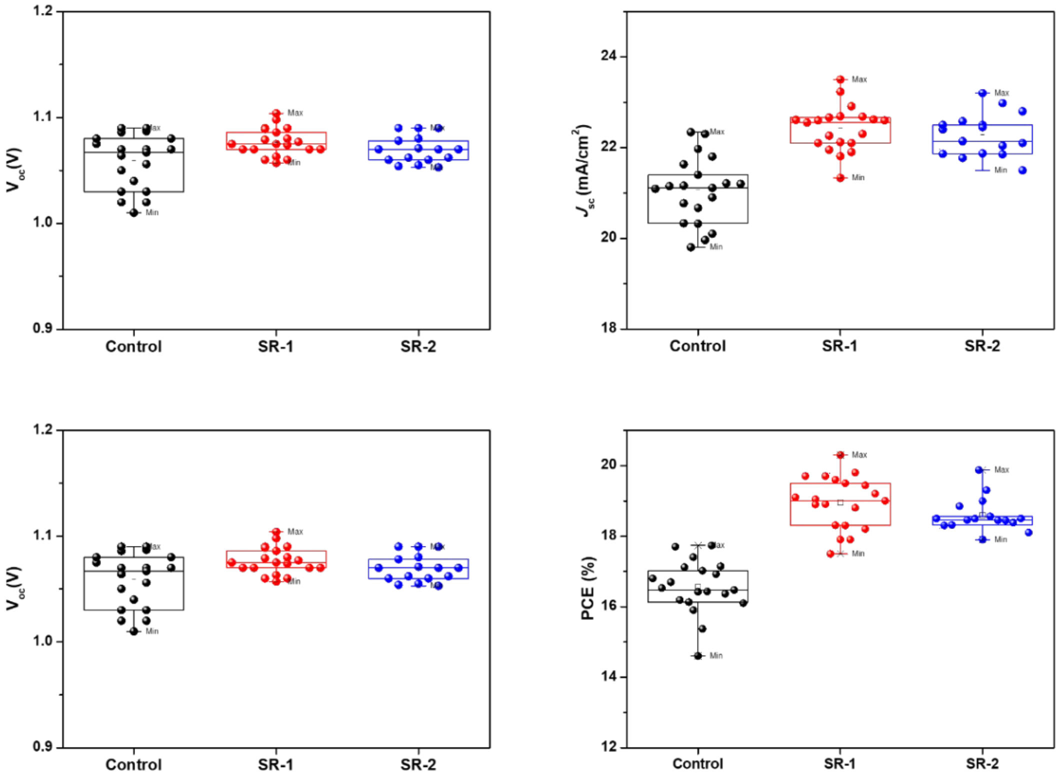

Figure S23. Statistics of the photovoltaic performance parameters with SR-1 (20 devices), SR-2 (16 devices), and control device (20 devices).

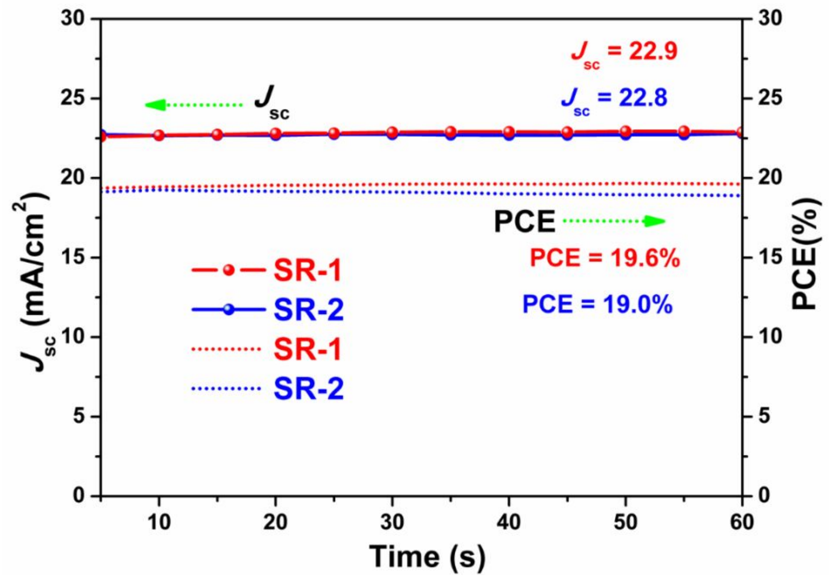

Figure S24. Steady-state photocurrent and output PCE at the maximum power point (MPP) of PSCs based on SR-1(red curve) and SR-2 (blue curve).

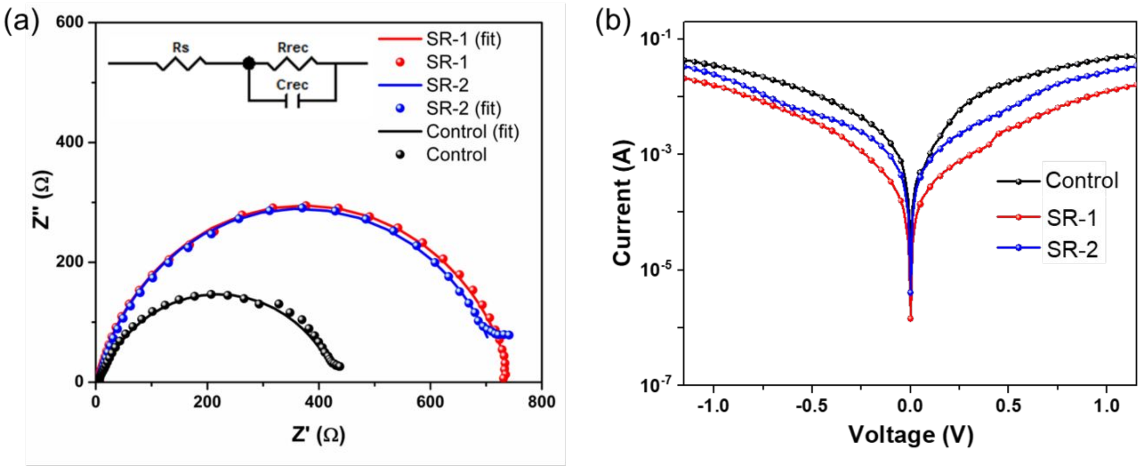

Figure S25. (a) Nyquist plots of control and SR-passivated devices, (solid line = fitted with experimental data; symbols = experimental data). (b) Dark current-voltage (*J-V*) curve of devices with the control, SR-1, and SR-2 films.

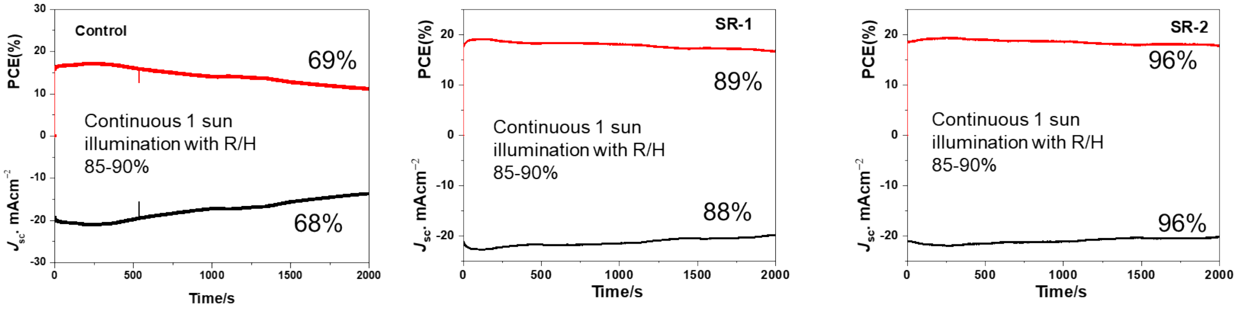

Figure S26. Stability of the devices under continuous illumination (AM1.5G) with MPPT at 85–90% relative humidity.

References

S1. Chiu, Y.-L.; Li, C.-W.; Kang, Y.-H.; Lin, C.-W.; Lu, C.-W.; Chen, C.-P.; Chang, Y. J., Dual-functional enantiomeric compounds as hole-transporting materials and interfacial layers in perovskite solar cells. *ACS Appl. Mater. Interfaces* **2022**, *14*, 26135-26147.

S2. Pangborn, A. B.; Giardello, M. A.; Grubbs, R. H.; Rosen, R. K.; Timmers, F. J. Safe and Convenient Procedure for Solvent Purification. *Organometallics* **1996**, *15*, 1518–1520.

S3. Lin, Y.-S.; Abate, S. Y.; Wang, C.-I.; Wen, Y.-S.; Chen, C.-I.; Hsu, C.-P.; Chueh, C.-C.; Tao, Y.-T.; Sun, S.-S., Low-cost hole-transporting materials based on carbohelicene for high-performance perovskite solar cells. *ACS Appl. Mater. Interfaces* **2021**, *13*, 20051-20059.

S4. Nakka, L.; Cheng, Y.; Aberle, A. G.; Lin, F., Analytical review of spiro-OMeTAD hole transport materials: paths toward stable and efficient perovskite solar cells. *Adv. Energy Sustainability Res.* **2022**, *3*, 2200045.

S5. Ok, S. A.; Jo, B.; Somasundaram, S.; Woo, H. J.; Lee, D. W.; Li, Z.; Kim, B.-G.; Kim, J. H.; Song, Y. J.; Ahn, T. K.; Park, S.; Park, H. J. Management of Transition Dipoles in Organic

Hole-Transporting Materials under Solar Irradiation for Perovskite Solar Cells. *Nat. Commun.* **2018**, *9*, 4537.

S6. Li, Z.; Jo, B. H.; Hwang, S. J.; Kim, T. H.; Somasundaram, S.; Kamaraj, E.; Bang, J.; Ahn, T. K.; Park, S.; Park, H. J. Bifacial Passivation of Organic Hole Transport Interlayer for NiOx-Based P-i-n Perovskite Solar Cells. *Adv. Sci.* **2019**, *6*, 1802163.

S7. Akula, S. B.; Su, C.; Wang, Y.-T.; Tingare, Y. S.; Chen, B.-R.; Jheng, Y.-C.; Lin, Y.-J.; Lan, H.-C.; Chang, Y.-C.; Lekphet, W.; Li, W.-R. Novel Thieno-Imidazole Salt-Based Hole Transport Material for Dopant-Free, Efficient Inverted Perovskite Solar Cell Applications. *J. Power Sources* **2021**, *483*, 229177.

S8. Feng, J.-Y.; Lai, K.-W.; Shiue, Y.-S.; Singh, A.; Kumar, CH. P.; Li, C.-T.; Wu, W.-T.; Lin, J. T.; Chu, C.-W.; Chang, C.-C.; Su, C. Cost-Effective Dopant-Free Star-Shaped Oligo-Aryl Amines for High Performance Perovskite Solar Cells. *J. Mater. Chem. A* **2019**, *7*, 14209–14221.

S9. Xia, J.; Joseph, V.; Sutanto, A. A.; Balasaravanan, R.; Ezhumalai, Y.; Zhang, Z.-X.; Ni, J.-S.; Tingare, Y. S.; Yau, S.-L.; Shao, G.; Qiu, Z.; Asiri, A. M.; Chen, M.-C.; Nazeeruddin, M. K. Isomeric Imidazole Functionalized Bithiophene-Based Hole Transporting Materials for Stable Perovskite Solar Cells and the Role of Donor Position. *Cell Rep. Phys. Sci.* **2022**, *4*, 101312.

S10. Akula, S. B.; Su, C.; Tingare, Y. S.; Lan, H.-C.; Lin, Y.-J.; Wang, Y.-T.; Jheng, Y.-C.; Lin, X.-C.; Chang, Y.-C.; Li, W.-R. Thieno-Imidazole Based Small Molecule Hole Transport Materials for Dopant-Free, Efficient Inverted (p–i–n) Perovskite Solar Cells. *J. Mater. Chem. C* **2020**, *8*, 16577–16583.

S11. Cheng, Y.; Fu, Q.; Zong, X.; Dong, Y.; Zhang, W.; Wu, Q.; Liang, M.; Sun, Z.; Liu, Y.; Xue, S. Coplanar Phenanthro[9,10-d]Imidazole Based Hole-Transporting Material Enabling over 19%/21% Efficiency in Inverted/Regular Perovskite Solar Cells. *Chem. Eng. J.* **2021**, *421*, 129823.

S12. Hua, M.; Luo, M.; Zong, X.; Cheng, Y.; Wang, T.; Wang, Z.; Li, C.; Sun, Y.; Xue, S. Configuration Engineering of Unsymmetrical Structural Hole Transport Material for Efficient and Stable Regular/Inverted Perovskite Solar Cells. *ACS Appl. Energy Mater.* **2023**, *6*, 9805–9814.

S13. Park, H.; Heo, J.; Jeong, B. H.; Lee, S.; Lee, K.-T.; Park, S.; Park, H. J. Interface Modification of Perovskite Solar Cell for Synergistic Effect of Surface Defect Passivation and Excited State Property Enhancement. *J. Alloys Compd*. **2023**, *960*, 170606.

S14. Tingare, Y. S.; Li, M. C.; Teng, S. H.; Lin, J. H.; Su, C.; Lin, S. J.; Lew, X. R.; Tsai, H.; Ghosh, D.; Nie, W., Charged Hole-Transporting Materials Based on Imidazolium for Defect Passivation in Inverted Perovskite Solar Cells. *Sol. RRL* **2024,** *8*, 2300817.

S15. Wang, H.; Zhang, W.; Wang, B.; Yan, Z.; Chen, C.; Hua, Y.; Wu, T.; Wang, L.; Xu, H.; Cheng, M., Modulating buried interface with multi-fluorine containing organic molecule toward efficient NiO_x-based inverted perovskite solar cell. *Nano Energy* **2023**, *111*, 108363.

S16. Hong, J.; Lee, Y. K.; Shin, S.; Whang, D. R.; Chang, D. W.; Park, H. J., Organic Interlayer for Enhanced Buried Interfaces in Wide-Bandgap Perovskite Solar Cells. *ChemSusChem* **2025**, *18*, e202500543.

S17. Chen, X. M.; Ye, Y. C.; Feng, S. C.; Lv, B. H.; Wang, J. Y.; Tang, J. X.; Dou, W. D., Improving buried interface contact by bidentate anchoring for inverted perovskite solar cells. *Small* **2024**, *20*, 2401256.

S18. Sun, X.; Zhang, C.; Gao, D.; Zhang, S.; Li, B.; Gong, J.; Li, S.; Xiao, S.; Zhu, Z.; Li, Z. a., Boosting Efficiency and Stability of NiO_x-Based Inverted Perovskite Solar Cells Through D–A Type Semiconductor Interface Modulation. *Adv. Funct. Mater.* **2024**, *34*, 2315157.

S19. Wang, Z.; Wang, Z.; Ma, Z.; Li, M.; Cheng, H.; Yang, Z.; Yan, S.; Zong, X.; Xiong, Y.; Jiang, Q., T-Shaped Linear Organic Semiconducting Interlayer Enables Highly Efficient NiO_x-Based Perovskite Solar Cells. *ACS Sustain. Chem. Eng.* **2025**, *13*, 13157-13165.

S20. Wang, X.; Jiang, J.; Hao, K.; Liu, Z.; Ge, H.; Cai, X.; Song, Y.; Yang, L.; Xu, H.; Li, A., Achieving Buried Interface/Bulk Synergistic Passivation via Chlorophyll Derivative for Efficient Inverted Perovskite Solar Cells. *Angew. Chem.* **2025**, *137*, e202504304.

S21. Fan, A.; An, M.; Zhang, T. K.; Fan, J. B.; Tian, H. R.; Du, P.; Chen, B. W.; Xia, J.; Huang, Y. K.; Fu, J. H., Corannulene-Derivative-Interfaced Inverted Perovskite Solar Cells with a Fill Factor above 0.87. *Adv. Funct. Mater.* **2025**, e10193. DOI: 10.1002/adfm.202510193

S22. Li, Z.; Sun, X.; Zheng, X.; Li, B.; Gao, D.; Zhang, S.; Wu, X.; Li, S.; Gong, J.; Luther, J. M., Stabilized hole-selective layer for high-performance inverted pin perovskite solar cells. *Science* **2023**, *382*, 284-289.

S23. Zhou, Y.; Huang, X.; Zhang, J.; Zhang, L.; Wu, H.; Zhou, Y.; Wang, Y.; Wang, Y.; Fu, W.; Chen, H., Interfacial modification of NiO_x for highly efficient and stable inverted perovskite solar cells. *Adv. Energy Mater.* **2024**, *14*, 2400616.

S24. Su, T.; Liu, W.; Xu, H.; Chen, H.; Wong, K. L.; Zhang, W.; Su, Q.; Wang, T.; Xu, S.; Liu, X., Self-assembled hole-transport material incorporating biphosphonic acid for dual-defect passivation in NiO_x-based perovskite solar cells. *J. Mater. Chem. A* **2024**, *12*, 33066-33075.

S25. Xu, Y.; Wang, C.; Amornkitbamrung, U.; Jeong, H. J.; Rhee, R. J. K.; In, Y.; Gibson, A.; Nakamura, T.; Truong, M. A.; Wakamiya, A., Molecular Bridge on Buried Interface for Energy Level Alignment in Inverted Perovskite Solar Cell with Efficiency over 25%. *ACS Energy Lett.* **2025**, *10*, 3407-3414.

S26. Gao, M.; Ou, Z.; Wang, C.; Liu, L.; Hu, D.; Wan, W.; Chen, P.; Pan, Y.; Nie, S.; Luo, Y., Multifunctional Buried Molecule-Bridge for High-Performance Inverted Perovskite Solar Cells. *Adv. Mater.* **2025**, e14273. DOI: 10.1002/adma.202514273

S27. Wang, J.; Jiao, B.; Tian, R.; Sun, K.; Meng, Y.; Bai, Y.; Lu, X.; Han, B.; Yang, M.; Wang, Y., Less-acidic boric acid-functionalized self-assembled monolayer for mitigating NiOx corrosion for efficient all-perovskite tandem solar cells. *Nat. Commun.* **2025**, *16*, 4148.

S28. Liu, Y.; Yang, X.; Ding, X.; Wang, J.; Xu, W.; Wang, X.; Zhang, L.; Yan, Y.; Wang, J.; Hou, Y., Fully Conjugated Co-Self-Assembled Monolayers for Efficient and Stable Inverted Perovskite Solar Cells. *Small* **2025**, *21*, 2502367.

S29. Xu, Z.; Sun, X.; Hui, W.; Wang, Q.; Xu, P.; Tang, W.; Hu, H.; Song, L.; Xu, X.; Wu, Y., Optimizing Molecular Packing and Interfacial Contact via Halogenated N-Glycidyl Carbazole Small Molecules for Low Energy Loss and Highly Efficient Inverted Perovskite Solar Cells. *Angew. Chem. Int. Ed.* **2025**, *24*, e202503008.

S30. Wang, Y.; Ju, H.; Mahmoudi, T.; Liu, C.; Zhang, C.; Wu, S.; Yang, Y.; Wang, Z.; Hu, J.; Cao, Y., Cation-size mismatch and interface stabilization for efficient NiO_x-based inverted perovskite solar cells with 21.9% efficiency. *Nano Energy* **2021**, *88*, 106285.

S31. Huang, Y.-J.; Cai, C.-E.; Feng, Y.-C.; Liu, B.-T.; Lee, R.-H., Water-Soluble Cationic Copolyacrylamides Modifying NiO_x for High-Performance Inverted Perovskite Solar Cells. *ACS Appl. Polym. Mater.* **2023**, *5*, 8949-8959.

S32. Yan, K.; Shen, Z.; Huang, Y.; Niu, B.; Chen, H.; Li, C.-Z., Curing the vulnerable heterointerface via organic-inorganic hybrid hole transporting bilayers for efficient inverted perovskite solar cells. *Chin. Chem. Lett.* **2024**, *35*, 109516.

S33. Tu, S.; Gang, Y.; Lin, Y.; Liu, X.; Zhong, Y.; Yu, D.; Li, X., Triple Cross-Linking Engineering Strategies for Efficient and Stable Inverted Flexible Perovskite Solar Cells. *Small* **2024**, *20*, 2310868.

S34. Chen, H.; Teale, S.; Chen, B.; Hou, Y.; Grater, L.; Zhu, T.; Bertens, K.; Park, S. M.; Atapattu, H. R.; Gao, Y.; Wei, M.; Johnston, A. K.; Zhou, Q.; Xu, K.; Yu, D.; Han, C.; Cui, T.; Jung, E. H.; Zhou, C.; Zhou, W.; Proppe, A. H.; Hoogland, S.; Laquai, F.; Filleter, T.; Graham, K. R.; Ning, Z.; Sargent, E. H., Quantum-size-tuned heterostructures enable efficient and stable inverted perovskite solar cells. *Nat. Photon.* **2022,** *16*, 352-358.

S35. Bai, S.; Da, P.; Li, C.; Wang, Z.; Yuan, Z.; Fu, F.; Kawecki, M.; Liu, X.; Sakai, N.; Wang, J. T.-W., Planar perovskite solar cells with long-term stability using ionic liquid additives. *Nature* **2019,** *571*, 245-250.

S36. Cui, X.; Jin, J.; Zou, J.; Tang, Q.; Ai, Y.; Zhang, X.; Wang, Z.; Zhou, Y.; Zhu, Z.; Tang, G., NiO_x nanocrystals with tunable size and energy levels for efficient and UV stable perovskite solar cells. *Adv. Funct. Mater.* **2022,** *32*, 2203049.

S37. Seo, S.; Jeong, S.; Bae, C.; Park, N. G.; Shin, H., Perovskite solar cells with inorganic electron-and hole-transport layers exhibiting long-term (≈ 500 h) stability at 85° C under continuous 1 sun illumination in ambient air. *Adv. Mater.* **2018,** *30*, 1801010.

S38. Kim, J. W.; Cho, E.; Lee, H. J.; Kwon, S. N.; Park, J. S.; Kim, M.; Kim, D. H.; Na, S. I.; Lee, S. J., Enhancing Efficiency of Inverted Perovskite Solar Cells by Sputtered Nickel Oxide Hole-Transport Layers. *Sol. RRL* **2024,** *8*, 2300933.

S39. Yang, Y.; Cheng, S.; Zhu, X.; Li, S.; Zheng, Z.; Zhao, K.; Ji, L.; Li, R.; Liu, Y.; Liu, C., Inverted perovskite solar cells with over 2,000 h operational stability at 85° C using fixed charge passivation. *Nat. Energy* **2024,** *9*, 37-46.

S40. Shen, G.; Dong, H.; Yang, F.; Ng, X. R.; Li, X.; Lin, F.; Mu, C., Application of an amphipathic molecule at the NiO_x/perovskite interface for improving the efficiency and long-term stability of the inverted perovskite solar cells. *J. Energy Chem.* **2023,** *78*, 454-462.

S41. Li, L.; Zhang, X.; Zeng, H.; Zheng, X.; Zhao, Y.; Luo, L.; Liu, F.; Li, X., Thermally-stable and highly-efficient bi-layered NiO_x-based inverted planar perovskite solar cells by employing a p-type organic semiconductor. *Chem. Eng. J.* **2022,** *443*, 136405.

S42. Gong, C.; Li, H.; Wang, H.; Zhang, C.; Zhuang, Q.; Wang, A.; Xu, Z.; Cai, W.; Li, R.; Li, X.; Zang, Z., Silver coordination-induced n-doping of PCBM for stable and efficient inverted perovskite solar cells. *Nat. Commun.* **2024**, *15*, 4922.

S43. Xu, Z.; Guo, Z.; Li, H.; Zhou, Y.; Liu, Z.; Wang, K.; Li, Z.; Wang, H.; Qaid, S. M. H.; Mohammed, O. F.; Zang, Z., Efficient and stable inverted MA/Br-free 2D/3D perovskite solar cells enabled by α-to-δ phase transition inhibition and crystallization modulation. *Energy Environ. Sci.* **2025**, *18*, 1354-1365.

S44. Park, S.; Kim, D. W.; Park, S. Y. Improved Stability and Efficiency of Inverted Perovskite Solar Cell by Employing Nickel Oxide Hole Transporting Material Containing Ammonium Salt Stabilizer. *Adv. Funct. Mater.* **2022**, *32*, 2200437.

S45. Zhang, J.; Yang, J.; Dai, R.; Sheng, W.; Su, Y.; Zhong, Y.; Li, X.; Tan, L.; Chen, Y. Elimination of Interfacial Lattice Mismatch and Detrimental Reaction by Self-Assembled Layer Dual-Passivation for Efficient and Stable Inverted Perovskite Solar Cells. *Adv. Energy Mater.* **2022**, *12*, 2103674.

S46. Chen, W.; Wu, Y.; Fan, J.; Djurišić, A. B.; Liu, F.; Tam, H. W.; Ng, A.; Surya, C.; Chan, W. K.; Wang, D.; et al. Understanding the Doping Effect on NiO: Toward High-Performance Inverted Perovskite Solar Cells. *Adv. Energy Mater.* **2018**, *8*, 1703519.

S47. Ge, B.; Lin, Z. Q.; Zhou, Z. R.; Qiao, H. W.; Chen, A. P.; Hou, Y.; Yang, S.; Yang, H. G. Boric Acid Mediated Formation and Doping of NiO Layers for Perovskite Solar Cells with Efficiency over 21%. *Sol. RRL* **2021**, *5*, 2000810.

S48. Parida, B.; Yoon, S.; Ryu, J.; Hayase, S.; Jeong, S. M.; Kang, D.-W. Boosting the Conversion Efficiency Over 20% in $MAPbI_3$ Perovskite Planar Solar Cells by Employing a Solution-Processed Aluminum-Doped Nickel Oxide Hole Collector. *ACS Appl. Mater. Interfaces* **2020**, *12*, 22958-22970.

S49. Ramanujam, R.; Hsu, H.-L.; Shi, Z.-E.; Lung, C.-Y.; Lee, C.-H.; Wubie, G. Z.; Chen, C.-P.; Sun, S.-S. Interfacial Layer Materials with a Truxene Core for Dopant-Free NiO_x-Based Inverted Perovskite Solar Cells. *Small* **2024**, *20*, 2310939.
